# Collection efficiencies of cylindrical and plane parallel ionization chambers: analytical and numerical results and implications for experimentally determined correction factors

**DOI:** 10.1088/1361-6560/ad63ed

**Published:** 2024-07-26

**Authors:** John D Fenwick, Sudhir Kumar, Juan Pardo-Montero

**Affiliations:** 1 Department of Medical Physics and Bioengineering, 8th Floor, Malet Place Engineering Building, University College London, Gower Street, London WC1E 6BT, England, United Kingdom; 2 Radiological Physics and Advisory Division, Bhabha Atomic Research Centre, CT & CRS Building, Anushaktinagar, Mumbai 400094, India; 3 Group of Medical Physics and Biomathematics, Instituto de Investigación Sanitaria de Santiago (IDIS), 15706 Santiago de Compostela, Spain; 4 Department of Medical Physics, Complexo Hospitalario Universitario de Santiago de Compostela, 15706 Santiago de Compostela, Spain

**Keywords:** ionization chamber, ion recombination, dose-per-pulse, collection efficiency, charge screening

## Abstract

*Objectives.* To derive a collection efficiency formula,$\,{f_{{\text{Gauss}}}}$, for cylindrical ionization chambers in pulsed radiation beams from a volume recombination model of Boag *et al* (1996 *Phys. Med. Biol.* **41** 885–97) including free electrons. To validate ${f_{{\text{Gauss}}}}$ and a parallel plate chamber formula ${f_{{\text{exp}}}}$ using an ion transport code and calculate changes in collection efficiencies caused by electric field charge screening at 0.1–100 mGy doses-per-pulse. And to determine collection efficiencies ${\text{C}}{{\text{E}}_{\infty} }$ predicted at infinite voltage in the absence of avalanche effects by fitting scaled formulae to efficiencies computed for 100–400 V chamber voltages and 10 and 100 mGy doses-per-pulse. *Approach.* Calculations were performed for an idealized parallel plate chamber with 2 mm electrode separation $d$, and for an idealized cylindrical chamber with 0.5 and 2.333 mm inner and electrode radii ${r_{{\text{in}}}}$ and ${r_{{\text{out}}}}$. *Main results.* ${f_{{\text{Gauss}}}}$ and ${f_{{\text{exp}}}}$ predict the same collection efficiencies for cylindrical and parallel plate chambers satisfying ${d^2} = \left( {r_{{\text{out}}}^2 - r_{{\text{in}}}^2} \right)\,\,{\text{ln}}\left( {{r_{{\text{out}}}}/{r_{{\text{in}}}}} \right)\,/\,2$, an equivalence condition met by the chambers studied. Without charge screening, efficiencies computed using the code equalled ${f_{{\text{Gauss}}}}$ and ${f_{{\text{exp}}}}$. With screening, efficiencies changed by ⩽0.03%, ⩽1.1% and ⩽21.3% at 1, 10 and 100 mGy doses-per-pulse, and differed between the chambers by ⩽0.9% and ⩽19.6% at ⩽10 and 100 mGy dose-per-pulse. For fits of ${f_{{\text{exp}}}}$ and ${f_{{\text{Gauss}}}}$, ${\text{C}}{{\text{E}}_{\infty} }$ values were ⩽1.2% and ⩽17.6% from unity at 10 and 100 mGy per pulse respectively, closer than for other formulae tested. *Significance.* Allowing for screening, ${f_{{\text{Gauss}}}}$ and ${f_{{\text{exp}}}}$ described computed collection efficiencies to within 0.03%, 1.1% and 21.3% at doses-per-pulse ⩽1, 10 and 100 mGy. Equivalence of the two chambers broke down at 100 mGy per pulse. Departures of ${\text{C}}{{\text{E}}_{\infty} }$ values from unity suggest that collection efficiencies determined experimentally by fitting ${f_{{\text{Gauss}}}}$ or ${f_{{\text{exp}}}}$ to readings made at multiple voltages will be accurate to within 1.2% and 17.6% at 10 and 100 mGy per pulse respectively.

## 1. Introduction

Doses-per-pulse are typically around 0.3 mGy in photon beams generated for radiotherapy (RT) treatments by linear accelerators (linacs) with flattening filters. In comparison, doses-per-pulse delivered by modern filter-free linacs, intra-operative electron beams and novel FLASH electron systems are respectively factors of 3, 30–300 and 30–15 000 higher. These larger doses-per-pulse lead to increased levels of ion recombination and reduced charge collection in radiation detectors, and this has renewed and intensified interest in recombination effects in dosimetry (Di Martino *et al* 2005, Laitano *et al* 2006, Christensen *et al* 2016, Petersson *et al* 2017, Gotz *et al* 2017, McManus *et al* 2020, Kranzer *et al* 2021).

Boag (1950) formulated a model of volume recombination between uniform clouds of positive and negative ions generated by a pulse of radiation, as they drift towards the cathode and anode of a parallel plate ionization chamber. From the model Boag obtained a formula *f* for chamber collection efficiency, defined as the ratio of charge collected to charge generated within the chamber. He also derived a geometric condition for equivalent recombination in cylindrical and parallel plate ionization chambers (Boag 1950, Boag and Currant 1980). Specifically, if the inner and outer electrode radii *r*_in_ and *r*_out_ of a cylindrical chamber and the electrode gap *d* of a parallel plate chamber satisfy the condition

\begin{equation*}\frac{1}{2}\,\left( {r_{{\text{out}}}^2 - r_{{\text{in}}}^2} \right)\,\,{\text{ln}}\left( {{r_{{\text{out}}}}/{r_{{\text{in}}}}} \right) = \,{d^2}\end{equation*}

then according to Boag’s 1950 model the collection efficiencies of the chambers should be the same when the same voltage is applied to each. If correct, this relationship allows collection efficiencies calculated for parallel plate ionization chambers to be carried across to equivalent cylindrical chambers, which is practically important since cylindrical chambers are used more often in RT dosimetry.

Subsequently, Boag *et al* (1996) extended the volume recombination model to account for free electron effects. The extended model describes the initial formation of uniform clouds of positive ions and free electrons, followed by rapid collection at the anode of a fraction *p* of the electrons and capture of the rest by oxygen molecules, forming a non-uniform cloud of negative ions. The negative ions drift towards the anode and are either collected there or recombine with positive ions first. Three approximate solutions of this model were derived for parallel plate chambers, together with associated collection efficiency formulae $f^{\prime}$, $f^{{\prime} {\prime}}$ and $f^{{\prime} {\prime} ^{\prime}}$. These approximate formulae predict collection efficiencies larger than those given by Boag’s 1950 *f* formula which excluded free electron effects.

Recently, the extended model was solved exactly for parallel plate chambers, across which the initial negative ion density varies exponentially (Fenwick and Kumar 2023). A collection efficiency formula *f*_exp_ was obtained that gave efficiencies in agreement with values calculated using a numerical code describing ion transport and recombination. Here we solve the same model exactly for a cylindrical geometry, obtaining a formula ${f_{{\text{Gauss}}}}$ for the collection efficiency of cylindrical chambers, in which the initial negative ion density varies according to a Gaussian function. We also derive three approximate collection efficiency formulae $f_{{\text{cyl}}}^{\prime}$, $f_{{\text{cyl}}}^{{\prime} {\prime}}$ and $f_{{\text{cyl}}}^{{\prime} {\prime} ^{\prime}}$ analogous to the three approximate formulae derived by Boag *et al* (1996) for the parallel plate geometry.

From the ${f_{{\text{Gauss}}}}$ and *f*_exp_ formulae we identify a condition for parallel plate and cylindrical chambers to have the same collection efficiencies, and we compare it to the condition found by Boag *et al* (1950) from his recombination model which excluded free electron effects.

The analytical development of recombination models provides more general results than can be obtained numerically. For models that include free electron effects, however, it involves two simplifications. First, it ignores screening of the electric field *E* within ionization chambers due to charge imbalances (ICRU 1982). This simplification is present in the derivations of collection efficiency formulae in this study and those of Boag *et al* (1996) and Fenwick and Kumar (2023). And second, it treats the rate-constant *γ* for attachment of free electrons to oxygen molecules as being constant throughout a chamber. For parallel plate chambers this is largely valid, but in cylindrical chambers the electric field strength varies with distance from the chamber axis, and *γ* is known to depend on *E* (Hochhäuser *et al* 1994, Boissonnat 2015).

To determine the effect of these additional factors, we calculate collection efficiencies for chambers using a numerical code that describes ion transport in the presence of *E*-field charge screening and *γ*(*E*) variation. As the derivation of ${f_{{\text{Gauss}}}}$ is lengthy the code is initially used with charge screening and *γ*(*E*) variation turned off, to check the formula in the conditions for which it was derived. Then we study by how much computed collection efficiencies change when *γ*(*E*) variation and charge screening are turned on in a cylindrical chamber, initially adding *γ*(*E*) variation and then screening to explore the relative impact of each factor. We also calculate changes in efficiencies computed for parallel plate chambers when screening is turned on. Further, we determine how well the analytical formulae describe the collection efficiencies computed with these effects turned on, and whether the equivalence condition between parallel plate and cylindrical chambers remains the same.

The code is also used to assess the accuracy of a common experimental method for determining chamber collection efficiencies, in which readings are taken at multiple chamber voltages and analyzed using recombination models (Boag 1950, Boag and Currant 1980, Boag *et al* 1996, Bruggmoser *et al* 2018a, Kranzer *et al* 2021). These multi-voltage methods relate the chamber reading ${R_{D,V}}$ obtained for a fixed dose *D* at voltage *V* to the collection efficiency at that voltage CE(*V*) via

\begin{equation*}{R_{D,V}} = {R_{D,\infty }} \times {\text{CE}}\left( V \right).\end{equation*}

Here ${R_{D,\infty }}$ is the reading in the absence of recombination, which is estimated by fitting readings made at practical voltages using the expression ${R_{D,\infty }} \times {\text{C}}{{\text{E}}_{{\text{model}}}}\left( V \right)$ in which ${\text{C}}{{\text{E}}_{{\text{model}}}}\left( V \right)$ is a closed-form collection efficiency formula such as *f* (Boag 1950). Given a fitted ${R_{D,\infty }}$ value, collection efficiencies can be calculated directly from readings ${R_{D,V}}$ using equation (2).

The accuracy of this approach is governed by the accuracy of the fitted ${R_{D,\infty }}$ value. In the final part of this study, we use a technique described in the *materials and methods* to assess the accuracy of ${R_{D,\infty }}$ estimation, working from collection efficiencies obtained from the ion transport code. Efficiencies calculated with and without *E*-field screening and $\gamma \left( E \right)$ variation are analyzed to characterize the impact of these effects on the experimental estimation of collection efficiencies. Several formulae are used for the ${\text{C}}{{\text{E}}_{{\text{model}}}}\left( V \right)$ term in the multi-voltage fitting process, and we investigate which performs best.

To make the study as accessible as possible, physical models of charge transport and recombination in ionization chambers are summarized in the *materials and methods*, together with approaches used in the derivation and analysis of collection efficiency formulae. The derivations are set out in the appendix, and efficiencies obtained from the formulae and numerical code are presented and analyzed in the *results*.

## 2. Materials and methods

### 2.1. Nomenclature

Quantities appearing outside the appendix are denoted by the following symbols—

*t,* time after a radiation pulse

*t_e_,* time by which no free electrons remain in an ionization chamber

*V*, potential difference across a chamber

$\underset{\raise0.3em\hbox{$\smash{\scriptscriptstyle-}$}}{E} $, $E,\,$ electric field and field strength

$x$, distance from the cathode towards the anode of a parallel plate chamber

*d*, electrode spacing in a parallel plate chamber

*r*, radius of a circle centred on the long axis of a cylindrical chamber

*r*_in_, *r*_out_, radii of cylindrical chamber inner and outer electrodes

*γ*, rate-constant for attachment of free electrons to oxygen

*v_e_,* drift velocity of free electrons

*k_1_, k_2_, k_e_*, mobilities of positive and negative ions and electrons

*a*, drift coefficient for free electrons

*p*, fraction of free electrons collected at the anode

$\lambda = \left( {1 - \sqrt {1 - p} } \right)$, dimensionless parameter

*n_0_*, initial uniform density of positively charged ions or free electrons

*n_e_*, density distribution of free electrons

${n_ \pm }$, density distributions of positively and negatively charged ions

*α*, rate-constant for recombination between positive and negative ions

*e*, electronic charge

${\varepsilon _{{\text{air}}}}$, permittivity of air at 20 °C and 101.325 kPa

$u = \frac{{{n_0}\alpha {g^2}}}{{\left( {{k_1} + {k_2}} \right)V}}$, dimensionless combination of parameters

$\Delta = \frac{\gamma }{{2{k_e}V}}\left( {r_{{\text{out}}}^2 - r_{{\text{in}}}^2} \right){\text{ ln}}\left( {{r_{{\text{out}}}}/{r_{{\text{in}}}}} \right)$, dimensionless combination of parameters

*g^2^*, geometric factor equal to *d^2^* or $\frac{1}{2}\left( {r_{{\text{out}}}^2 - r_{{\text{in}}}^2} \right)\,{\text{ln}}\left( {{r_{{\text{out}}}}/{r_{{\text{in}}}}} \right)$

${f_{{\text{logist}}}}$, empirical ionization chamber collection efficiency formula

$f$, Boag ionization chamber collection efficiency without free electron effects

$f^{\prime}$, $f^{{\prime} {\prime}}$, $f^{{\prime} {\prime} ^{\prime}}$, ${f_{{\text{exp}}}}$, parallel plate chamber efficiency formulae with free electrons

$f_{{\text{cyl}}}^{\prime}$, $f_{{\text{cyl}}}^{{\prime} {\prime}}$, $f_{{\text{cyl}}}^{{\prime} {\prime} ^{\prime}}$, ${f_{{\text{Gauss}}}}$, cylindrical chamber efficiency formulae with free electrons

${R_{D,V}}$, chamber reading for dose *D* at voltage *V*

${R_{D,\infty }}$, multi-voltage estimate of chamber reading with no recombination

${\text{C}}{{\text{E}}_{{\text{model}}}}\left( V \right)$, model of collection efficiency variation with voltage

${\text{C}}{{\text{E}}_{\infty} }$, estimate of collection efficiency at *V* $ \to \infty \,$from multi-voltage data fit

*E_1_*, exponential integral function

The polarity of cylindrical ionization chambers is described as positive/negative when the central electrode is at a higher/lower potential than the outer one and forms the anode/cathode. A glossary in the appendix lists further key symbols used there.

### 2.2. Charge transport and ion recombination models

In Boag’s 1950 model, a radiation pulse generates uniform clouds of free electrons and positively charged ions, both with initial number density *n_0_*. The electrons rapidly attach to oxygen molecules, forming a uniform cloud of negatively charged ions also of density *n_0_*. Subsequently the positive and negative ions drift towards the cathode and anode, with average speeds given by their mobilities *k_1_* and *k_2_* multiplied by the local electric field strength *E*. During the drift the positive and negative ions recombine at a rate given by the product of their local densities and the rate-constant *α.* The resulting collection efficiency $f$ works out as

\begin{equation*}f\, = \,\frac{1}{u}{\text{ln}}\left( {1 + u} \right)\, \approx \,1 - u/2\,{\text{for}}\,u \ll 1\end{equation*}

\begin{equation*}\kern-14.6pc{\text{where}}\qquad\qquad\qquad\qquad\qquad\qquad\qquad\qquad\;u\, = \frac{{{n_0}\alpha }}{{\left( {{k_1} + {k_2}} \right)V}}\,{g^2}\end{equation*}

and *g^2^* is given by ${d^2}$ for parallel plate chambers and $\frac{1}{2}\,\left( {r_{{\text{out}}}^2 - r_{{\text{in}}}^2} \right)\,\,{\text{ln}}\left( {{r_{{\text{out}}}}/{r_{{\text{in}}}}} \right)$ for cylindrical chambers. The model collection efficiency falls with initial ion density and electrode separation but rises with the potential difference *V* across the chamber.

In the extended model of Boag *et al* (1996) the free electrons drift towards the anode with an average speed *v_e_* given by their mobility *k_e_* multiplied by the electric field strength *E*. Electron drift speeds are much faster than those of ions, and consequently in analytical models the electrons are considered to have either been collected at the anode or become attached to oxygen almost instantly, by time *t*≈ 0 following a radiation pulse, before the positive ions have moved from the locations in which they were generated.

The attachment rate is the product of the local electron density *n_e_* and the rate-constant *γ*, from which it follows that in a parallel plate chamber the fraction *p* of electrons reaching the anode is

\begin{equation*}p = \frac{{\left( {1 - {\text{exp}}\left( { - ad} \right)} \right)}}{{ad}} = \,\frac{{{k_e}V}}{{\gamma {d^2}}}\,\left\{ {1 - {\text{exp}}\left( { - \frac{{\gamma {d^2}}}{{{k_e}V}}} \right)} \right\}\,.\end{equation*}

The drift constant, *a*, in equation (5) is the attachment rate-constant per distance travelled by the electrons, equal to $\left( {\gamma /{v_e}} \right)$ and thus $\left( {\gamma d/\left( {{k_e}V} \right)} \right)$ since the *E*-field strength in the chamber is *V*/*d*. The electrons that attach to oxygen form a cloud of negative ions, which in a parallel plate chamber has the initial density distribution

\begin{equation*}{n_ - }\left( {x,t = 0} \right)\, = \,{n_0}\,\left( {1 - {\text{exp}}\left( { - ax} \right)} \right)\end{equation*}

where $x$ denotes distance from the cathode (Boag *et al* 1996). The non-uniformity of this distribution makes it more difficult to solve the ion recombination equations. Consequently, Boag *et al* (1996) obtained collection efficiency formulae for parallel plate chambers based on three approximations to the distribution of equation (6) which describe uniform density levels—

• *n_0_* (1-*p*) throughout the ionization chamber;
• *n_0_* throughout a region distant *pd*→*d* from the cathode, and zero elsewhere;
• ${n_0}\left( {1 - \lambda } \right)$ in the region $\lambda d$→$d$ from the cathode where $\lambda = \left( {1 - \sqrt {1 - p} } \right)$, and zero elsewhere.

For these initial negative ion distributions, Boag *et al* (1996) derived the approximate collection efficiency formulae

\begin{equation*}f{^{{\prime}}} = \,\frac{1}{u}{\text{ln}}\left\{ {1 + \frac{{{\text{exp}}\left( {pu} \right) - 1}}{p}\,} \right\}\end{equation*}

\begin{equation*}f{^{{\prime} {\prime}}} = \,p + \frac{1}{u}{\text{ ln}}\,\left\{ {1 + u\left( {1 - p} \right)} \right\}\end{equation*}

\begin{equation*}f{^{{\prime} {\prime} ^{\prime}}} = { }\lambda + \frac{1}{u}{\text{ln}}\left\{ {{ }1 + \frac{{{\text{exp}}\left( {\lambda \left( {1 - \lambda } \right)u} \right) - 1}}{\lambda }{ }} \right\}.\end{equation*}

Whereas $f$ depends only on the composite parameter $u$, these formulae additionally depend on *p* and thus $ad$. Across the ranges *u* = 0.01–0.50 and *p*= 0.1–0.6, Boag *et al* (1996) found that differences between collection efficiencies predicted by the three approximate formulae reached 5.1% relative.

Solving the extended model exactly for a parallel plate chamber with the initial exponential negative ion distribution of equation (6) instead gives a collection efficiency formula (Fenwick and Kumar 2023)

\begin{equation*}{f_{{\text{exp}}}} = \frac{1}{u}\,\ln \,\left\{ {1 + u\,h\left( {u,ad} \right)} \right\}\end{equation*}

where

\begin{equation*}h\left( {u,ad} \right) = \,\frac{1}{{ad}}{\text{exp}}\left( {\frac{u}{{ad}}} \right)\left\{ {\,{E_1}\left( {\frac{u}{{ad}}{\text{exp}}\left( { - ad} \right)} \right) - {E_1}\left( {\frac{u}{{ad}}} \right)\,} \right\}\end{equation*}

and *E*_1_ is the standard exponential integral function (Gradshteyn and Rhyzik 2007) for which tabulated values are widely available.

Values of electron and ion mobilities *k_e_, k*_1_, *k*_2_ used in this study are listed in table 1, together with the electron attachment and ion recombination rate-constants $\gamma $ and $\alpha $. They are derived from data compiled by Gotz *et al* (2017). The table also includes a formula describing the variation of *γ* with *E*, a value for the permittivity of air, ${\varepsilon _{{\text{air}}}}$, and an *n*_0_-per-Gy value which when multiplied by the dose-per-pulse gives the initial density *n*_0_ of free electrons and positive ions initially generated by a radiation pulse.

**Table 1.** Values of electron and ion mobilities and rate-constants of electron attachment and ion recombination used in collection efficiency calculations. The number of free electrons liberated per Gy and the permittivity of dry air are also listed.

Parameter	Value^a^
*k_e_*	8.3 × 10^−2^ m^2^V^−1^s^−1^
*k_1_*	1.87 × 10^−4^ m^2^V^−1^s^−1^
*k_2_*	2.09 × 10^−4^ m^2^V^−1^s^−1^
*γ*	[1.1 + 11.3 × exp(−1.04 × 10^−5^ × *E* ^b^)] × 10^7^ s^−1^ for *E* ⩾0.327 × 10^5^ V m^−1^
	7.0 × 10^7^ + 657 × *E* s^−1^ for *E* < 0.327 × 10^5^ V m^−1^
*α*	1.30 × 10^−12^ m^3^s^−1^
*n*_0_-per-Gy	2.22 × 10^17^ m^−3^Gy^−1^
${\varepsilon _{{\text{air}}}}$	8.86 × 10^−12^ m^−3^kg^−1^s^4^A^2^

^a^ All values derived from Gotz *et al* (2017) except for *n_0_-*per-Gy, the initial free electron density per Gy, which follows directly from (*W*/*e*) and the density of dry air at 20 °C and 101.325 kPa, and ${\varepsilon _{air}}$ which is the permittivity of air at the same temperature and pressure.

^b^ *E* is electric field-strength measured in V m^−1^.

### 2.3. Collection efficiency derivations for the extended model in a cylindrical geometry

**Figure 1.**
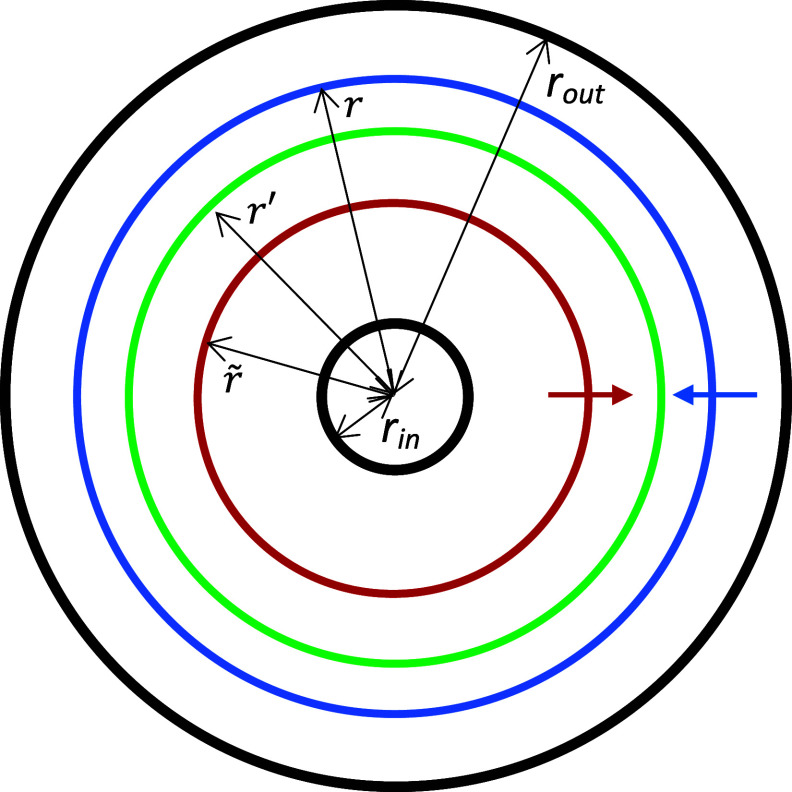
Schematic cross-section of a cylindrical ionization chamber, showing inner and outer electrodes (black) with radii *r_in_* and *r_out_*. Annuli of ions of opposite sign are also shown moving inwards and outwards (blue and red). In the exact derivation of collection efficiencies presented in the appendix, $r$ and $\tilde r$ denote the radial locations at which these ions are formed, while $r^{\prime}$ (green) denotes the radius at which ions belonging to the two annuli cross over as they drift to the electrodes. In the figure, $r^{\prime}$ lies closer to $r$ than to $\tilde r$. This represents the situation when the ions drifting inwards are positive, since these ions have ∼20% lower mobilities than do negative ions (table 1).

Figure 1 shows a schematic cross-section through a cylindrical ionization chamber. The chamber is idealized as being infinitely long rather than having a finite length like practical chambers which employ guard-rings to retain electric field uniformity towards the chamber ends. The same geometry was studied by Boag (1950). The parallel plate chamber geometry studied in this work is similarly idealized as comprising electrodes with infinite cross-sectional area but narrow separation, again reflecting the approach taken by Boag (1950) and Boag *et al* (1996).

Collection efficiency derivations for the cylindrical geometry are detailed in the appendix. Several times we invoke a counter-intuitive deduction of Boag (1950), that densities of ions travelling towards the inner or outer electrodes of cylindrical ionization chambers do not change with radial distance from the chamber axis, $r^{\prime}$, except due to ion recombination. Clearly, the circumferential length occupied by the thin annulus of ions shown travelling towards the central electrode in figure 1 falls with decreasing $r^{\prime}$. However, the variation of *E* with $r^{\prime}$ causes the leading edge of the ions to move inward faster than the outer edge. This increases the radial thickness of the annulus throughout which the ions are spread, to a degree that exactly offsets the fall in circumferential length.

The gradual broadening of the annulus of ions might be expected to cause it to begin overlapping neighbouring annuli. This would invalidate Boag’s deduction but does not in fact happen. Consider two neighbouring annuli both moving towards the centre of the chamber, the inner one slightly faster on average. Since the ions at the outer edge of the inner annulus and the inner edge of the outer annulus experience the same *E*-field and therefore move at the same average speed, the two annuli neither overlap nor pull apart as they drift towards the central electrode, despite both widening.

### 2.4. Electric field calculations and the numerical code

In the absence of charge imbalance, the electric field in a parallel plate chamber runs along the $x$-axis perpendicular to the planes of the electrodes with magnitude $\left( {V/d} \right)$, whereas the field in a cylindrical chamber runs radially with magnitude $\left( {V/\left( {r\,{\text{ln}}\left( {{r_{{\text{out}}}}/{r_{{\text{in}}}}} \right)} \right)} \right)$.

These fields were used in the analytical derivations of collection efficiency formulae. However, charge imbalances do exist between positive ions and free electrons generated directly by a radiation pulse and the negative ions formed by attachment of electrons to oxygen. These imbalances partly screen the electric field, which is further modified as the ions drift towards the electrodes (ICRU 1982). Boag *et al* (1996) have estimated that errors in calculated collection efficiencies due to omission of screening effects should be small at doses-per-pulse up to 10 mGy, though more substantial at ⩾100 mGy.

From Gauss’s law, the electric fields in the presence of charge imbalance are described by

\begin{equation*}\underset{\raise0.3em\hbox{$\smash{\scriptscriptstyle-}$}}{\nabla } .\underset{\raise0.3em\hbox{$\smash{\scriptscriptstyle-}$}}{E} = {\text{d}}{E_x}/{\text{d}}x = \left( {{n_ + }\left( x \right) - {n_ - }\left( x \right) - {n_e}\left( x \right)} \right){ }e/{\varepsilon _{{\text{air}}}}{ }\end{equation*}

for parallel plate ionization chambers and

\begin{equation*}\underset{\raise0.3em\hbox{$\smash{\scriptscriptstyle-}$}}{\nabla } .\underset{\raise0.3em\hbox{$\smash{\scriptscriptstyle-}$}}{E} = \frac{1}{r}\frac{{\text{d}}}{{{\text{d}}r}}\left( {rE\left( r \right)} \right) = { }\frac{E}{r} + \frac{{{\text{d}}E\left( r \right)}}{{{\text{d}}r}} = \left( {{n_ + }\left( r \right) - {n_ - }\left( r \right) - {n_e}\left( r \right)} \right){ }e/{\varepsilon _{{\text{air}}}}{ }\end{equation*}

for cylindrical chambers (Gotz *et al* 2017, Kranzer *et al* 2021). Given the densities of positive and negative ions and electrons ${n_ + }$, ${n_ - }$ and ${n_e}$, the electronic charge *e* and the boundary condition that the path integral of $\underset{\raise0.3em\hbox{$\smash{\scriptscriptstyle-}$}}{E} $ across a chamber equals *V,* equations (12) and (13) can be solved numerically. Thus, the coupled charge transport and electric field evolution process can be computed using a repeating code in which the charge drift and recombination over a short period of time are calculated for a previously computed *E*-field, then *E* is updated to reflect the resulting change in charge distribution.

We implemented such a code in MATLAB R2022b (Mathworks, Natick, MA), adapting a previous program (Pardo *et al* 2005) to include free electrons. At early time-points the rapid drift of free electrons and much slower drift of ions were computed together, whereas in analytical derivations of collection efficiency formulae they are typically considered entirely sequentially. Transport and recombination were modelled thus

\begin{equation*}\frac{{\partial {n_e}\left( {\underset{\raise0.3em\hbox{$\smash{\scriptscriptstyle-}$}}{s} ,t} \right)}}{{\partial t}} = \, - \gamma {n_e}\left( {\underset{\raise0.3em\hbox{$\smash{\scriptscriptstyle-}$}}{s} ,t} \right) + {k_e}\underset{\raise0.3em\hbox{$\smash{\scriptscriptstyle-}$}}{\nabla } .\left( {{n_e}\left( {\underset{\raise0.3em\hbox{$\smash{\scriptscriptstyle-}$}}{s} ,t} \right)\,\underset{\raise0.3em\hbox{$\smash{\scriptscriptstyle-}$}}{E} \left( {\underset{\raise0.3em\hbox{$\smash{\scriptscriptstyle-}$}}{s} ,t} \right)} \right)\end{equation*}

\begin{equation*}\frac{{\partial {n_ - }\left( {\underset{\raise0.3em\hbox{$\smash{\scriptscriptstyle-}$}}{s} ,t} \right)}}{{\partial t}} = \, + \gamma {n_e}\left( {\underset{\raise0.3em\hbox{$\smash{\scriptscriptstyle-}$}}{s} ,t} \right) + {k_ - }\underset{\raise0.3em\hbox{$\smash{\scriptscriptstyle-}$}}{\nabla } .\left( {{n_ - }\left( {\underset{\raise0.3em\hbox{$\smash{\scriptscriptstyle-}$}}{s} ,t} \right)\,\underset{\raise0.3em\hbox{$\smash{\scriptscriptstyle-}$}}{E} \left( {\underset{\raise0.3em\hbox{$\smash{\scriptscriptstyle-}$}}{s} ,t} \right)} \right) - \alpha \,{n_ - }\left( {\underset{\raise0.3em\hbox{$\smash{\scriptscriptstyle-}$}}{s} ,t} \right)\,{n_ + }\left( {\underset{\raise0.3em\hbox{$\smash{\scriptscriptstyle-}$}}{s} ,t} \right)\end{equation*}

\begin{equation*}\frac{{\partial {n_ + }\left( {\underset{\raise0.3em\hbox{$\smash{\scriptscriptstyle-}$}}{s} ,t} \right)}}{{\partial t}} = \, - {k_ - }\,\underset{\raise0.3em\hbox{$\smash{\scriptscriptstyle-}$}}{\nabla } .\left( {{n_ + }\left( {\underset{\raise0.3em\hbox{$\smash{\scriptscriptstyle-}$}}{s} ,t} \right)\,\underset{\raise0.3em\hbox{$\smash{\scriptscriptstyle-}$}}{E} \left( {\underset{\raise0.3em\hbox{$\smash{\scriptscriptstyle-}$}}{s} ,t} \right)} \right) - \alpha \,{n_ + }\left( {\underset{\raise0.3em\hbox{$\smash{\scriptscriptstyle-}$}}{s} ,t} \right){n_ - }\left( {\underset{\raise0.3em\hbox{$\smash{\scriptscriptstyle-}$}}{s} ,t} \right)\end{equation*}

where $\underset{\raise0.3em\hbox{$\smash{\scriptscriptstyle-}$}}{s} $ denotes a point is space and the equations reflect the processes described in *section 2.2*. The *E*-fields for parallel plate and cylindrical chambers were defined by equations (12) and (13) when considering screening, and by $\left( {V/d} \right)$ and $\left( {V/\left( {r\,{\text{ln}}\left( {{r_{{\text{out}}}}/{r_{{\text{in}}}}} \right)} \right)} \right)$ when screening was not considered.

Within the code, space was discretized in steps $\Delta x = \Delta r = 0.5\,\mu {\text{m}}$. The time-step $\Delta t$ was 1/25th of the time for the fastest particle being tracked to traverse $\Delta x$ or $\Delta r$. Thus

\begin{align*} \Delta t&amp; = \left( {\Delta x{\text{ or }}\Delta r} \right)\,/\,\left( {25\,{k_e}\,{\text{max}}\left( E \right)} \right)\,\,\,\,{\text{for}}\,\,\,t \unicode{x2A7D} {t_e}, \nonumber \\ &amp; = \left( {\Delta x{\text{ or }}\Delta r} \right)\,/\,\left( {25\,{k_2}\,{\text{max}}\left( E \right)} \right)\,\,\,\,{\text{for}}\,\,\,t &gt; {t_e} \end{align*}

where *t_e_* is the time by which no free electrons remain in the chamber, approximated as when the electron density everywhere has fallen to $ \unicode{x2A7D} {10^{ - 9}}\,{n_0}$. Following a time-step, each point’s particle density was updated to reflect transport and recombination. The transport component was accounted for by subtracting from the density at each point the difference in densities between that point and the adjacent point from which particles flow into it, multiplied by the ratio of the particle transport distance at that time-step to $\Delta x$ or ${ }\Delta r$. The code is available for download at http://github.com/juancho-pm/Code-for-Fenwick-et-al-PMB2024.

### 2.5. Collection efficiency calculations

Collection efficiencies were calculated for a parallel plate chamber with an electrode separation of 2 mm, and for a cylindrical chamber with inner and outer electrode radii of 0.5 mm and 2.333 mm. The electrode separation of the parallel plate chamber matches the classic Markus chamber (PTW 23 343, Freiburg, Germany), the NACP Plane Parallel chamber (IBA, Louvain-la-Neuve, Belgium) and the Exradin A10 (Standard Imaging, Madison, WI). The inner radius of the cylindrical chamber matches the Exradin A1SL and A12S chambers, the IBA CC13 and FC23-C chambers (Iwafuchi *et al* 2022), and standard Farmer chambers including the Exradin A12, IBA FC65-G and NE2571A (Phoenix Dosimetry Ltd, Sandhurst, UK). The outer radius was chosen so that the cylindrical and parallel chambers were equivalent according to the condition of equation (1). It lies between the 2 mm outer radius of the Exradin A1SL chamber and the 3–3.15 mm outer radii of the rest.

For both chambers, collection efficiencies were calculated for chamber potential differences of 200 and 400 V, covering the range of commonly used voltages. Doses-per-pulse of 0.1, 1, 10 and 100 mGy were chosen, covering the range delivered by linacs with and without flattening filters and intra-operative electron beams, and the lower part of the range delivered by electron FLASH systems. For the cylindrical chamber, calculations were made for positive and negative polarities. The ${f_{{\text{Gauss}}}}$ formula was initially checked against collection efficiencies obtained from the ion transport code run under the conditions for which the formula was derived. Specifically, the *E*-field strength was defined as $\left( {V/\left( {r\,{\text{ln}}\left( {{r_{{\text{out}}}}/{r_{{\text{in}}}}} \right)} \right)} \right)$ and the rate-constant *γ* throughout the cylindrical chamber was set to its uniform level in the equivalent parallel plate chamber with the same voltage applied, calculated from the $\gamma \left( E \right)$ formula in table 1 with *E* defined as $\left( {V/d} \right)$ = 500 × *V* Vm^−1^.

Next, the effect of *γ*(*E*) variation was tested by re-running the code for the cylindrical chamber, with *E* again defined ignoring charge screening but *γ* varying with *E* as described in table 1. Finally, the full code was run for parallel plate and cylindrical chambers to check for the effects of screening. This was done for the 200 and 400 V potential differences for which the preceding sets of collection efficiencies were calculated, and for 100, 300, 700 and 1000 V to further characterize the variation of collection efficiency with chamber voltage. Computed efficiencies were plotted using the *Origin* software (OriginLab, Northampton, MA) and we assessed which of the collection efficiency formulae best described the efficiency data computed under the different conditions.

### 2.6. Multi-voltage estimation of collection efficiencies

The accuracy with which ${R_{D,\infty }}$ is estimated by fitting ${R_{D,\infty }} \times {\text{CE}}\left( V \right)$ to readings made at multiple voltages *V* determines the accuracy of experimental multi-voltage methods for estimating collection efficiencies. It can be assessed by taking a series of collection efficiencies computed for different voltages, fitting them using $\left( {{\text{C}}{{\text{E}}_{\infty} } \times {\text{C}}{{\text{E}}_{{\text{model}}}}\left( V \right)} \right)$ where ${\text{C}}{{\text{E}}_{\infty} }$ is a fitted scaling factor, and finding how close the value of ${\text{C}}{{\text{E}}_{\infty} } \times {\text{C}}{{\text{E}}_{{\text{model}}}}\left( V \right)$ is to a collection efficiency of one in the absence of recombination at $V \to \infty $. Since the collection efficiency formulae used to describe ${\text{C}}{{\text{E}}_{{\text{model}}}}\left( V \right)$ in this study all take values of one at $V \to \infty $, this is a check of the relative difference between unity and the fitted factor ${\text{C}}{{\text{E}}_{\infty} }$. This is the same as the relative difference between the true value of ${R_{D,\infty }}$ and the value obtained by fitting ${R_{D,\infty }} \times {\text{C}}{{\text{E}}_{{\text{model}}}}\left( V \right)$ to measured readings ${R_{D,V}}$, since the readings are essentially collection efficiencies multiplied by the true ${R_{D,\infty }}$ value.

We therefore fitted ${\text{C}}{{\text{E}}_{\infty} } \times {\text{C}}{{\text{E}}_{{\text{model}}}}\left( V \right)$ to collection efficiencies computed for the parallel plate and positively and negatively polarized cylindrical chambers. The parallel plate data were fitted using the $f$, $f^{\prime}$, $f^{{\prime} {\prime}}$, $f^{{\prime} {\prime} ^{\prime}}$ and ${f_{{\text{exp}}}}$ formulae to describe ${\text{C}}{{\text{E}}_{{\text{model}}}}\left( V \right)$, and the cylindrical chamber data were fitted using the cylindrical equivalents. Along with ${\text{C}}{{\text{E}}_{\infty} }$, values of the *u* and *ad* parameters of the parallel plate formulae were fitted to achieve the best description of the data, rather than being defined as in equations (4) and (5) and determined by the chamber geometry and the transport and recombination coefficients listed in table 1. Parameters of the cylindrical chamber formulae were similarly fitted. We also used an empirical logistic collection efficiency formula developed by Petersson *et al* (2017) which has the form

\begin{equation*}{f_{{\text{logist}}}} = { }1/{\left( {1 + {{\left( {{\text{DPP}}/V} \right)}^\varepsilon }} \right)^\nu },\end{equation*}

where DPP is the dose-per-pulse measured in mGy and *ϵ* and *ν* are fitted parameters.

Fitting was carried out using the *nls* function and *nl2sol* algorithm in *R* version 4.0.2 (*R* Foundation, Vienna, Austria). Complete sets of collection efficiencies calculated for chamber voltages of 100–1000 V were fitted, as were subsets calculated for voltages ⩽400 V to test the performance of the multi-voltage approach over a practical range of chamber voltages.

Initially we fitted idealized collection efficiencies calculated using the numerical code with *γ*(*E*) variation and *E*-field charge screening turned off. For cylindrical chambers, we also fitted collection efficiencies calculated with *γ*(*E*) variation turned on. For parallel plate and cylindrical chambers, the process was repeated with charge screening also turned on.

## 3. Results

### 3.1. Exact and approximate collection efficiency formulae for cylindrical chambers

Derivations of collection efficiency formulae from the model of Boag *et al* (1996) are detailed in the appendix. For a cylindrical chamber with positive polarity, the initial density distribution of negative ions formed by free electrons is shown to vary with *r* according to a Gaussian function

\begin{equation*}{n_ - }\left( {r,\,t = 0} \right)\, = \,\,{n_0}\,\left\{ {\,1 - {\text{exp}}\left( { - \Delta \frac{{\left( {r_{{\text{out}}}^2 - \,{r^2}} \right){ }}}{{\left( {r_{{\text{out}}}^2 - \,r_{{\text{in}}}^2} \right)}}} \right)\,} \right\},\end{equation*}

\begin{equation*}\kern-13.2pc{\text{where }}\qquad\qquad\qquad\qquad\qquad\quad\Delta = { }\frac{\gamma }{{2{k_e}V}}\left( {r_{{\text{out}}}^2 - r_{{\text{in}}}^2} \right){\text{ ln}}\left( {{r_{{\text{out}}}}/{r_{{\text{in}}}}} \right){ }.\end{equation*}

For negative polarity, the negative ion density varies as

\begin{equation*}{n_ - }\left( {r,{ }t = 0} \right) = { }{n_0}\left\{ {1 - {\text{exp}}\left( { - \Delta \frac{{\left( {{r^2} - { }r_{{\text{in}}}^2} \right){ }}}{{\left( {r_{{\text{out}}}^2 - { }r_{{\text{in}}}^2} \right)}}} \right)} \right\},\end{equation*}

and in both cases the fraction of free electrons collected is

\begin{equation*}p = { }\left( {1 - {\text{exp}}\left( { - \Delta } \right)} \right)/\Delta .\end{equation*}

Thus *p* has the same form as for parallel plate chambers (equation (5)) but with $\Delta $ replacing *ad*, or equivalently $\left( {r_{{\text{out}}}^2 - r_{{\text{in}}}^2} \right)\,\,{\text{ln}}\left( {{r_{{\text{out}}}}/{r_{{\text{in}}}}} \right)/2$ replacing *d^2^*.

Given the initial negative ion density of equation (19), the collection efficiency formula obtained by exactly solving the extended recombination model for a positively polarized cylindrical ionization chamber is

\begin{equation*}{f_{{\text{Gauss}}}} = \frac{1}{u}{\text{ln}}\left\{ {\,1 + u\,h\left( {u,\Delta } \right)\,} \right\}.\end{equation*}

This has the same form as the ${f_{{\text{exp}}}}$ formula of equation (10) for parallel plate chambers, but in the *u* and $\Delta $ terms of equation (23) the *d^2^* factor that appears in the equivalent *u* and *ad* terms of equation (11) is replaced with $\frac{1}{2}{ }\left( {r_{{\text{out}}}^2 - r_{{\text{in}}}^2} \right){\text{ ln}}\left( {{r_{{\text{out}}}}/{r_{{\text{in}}}}} \right)$. Explicit formulae for $h\left( {u,\Delta } \right)$, $u$ and $\Delta $ are provided in equations (A37)–(A39) of the appendix. Collection efficiencies for negatively polarized cylindrical chambers are also described by equation (23).

**Figure 2.**
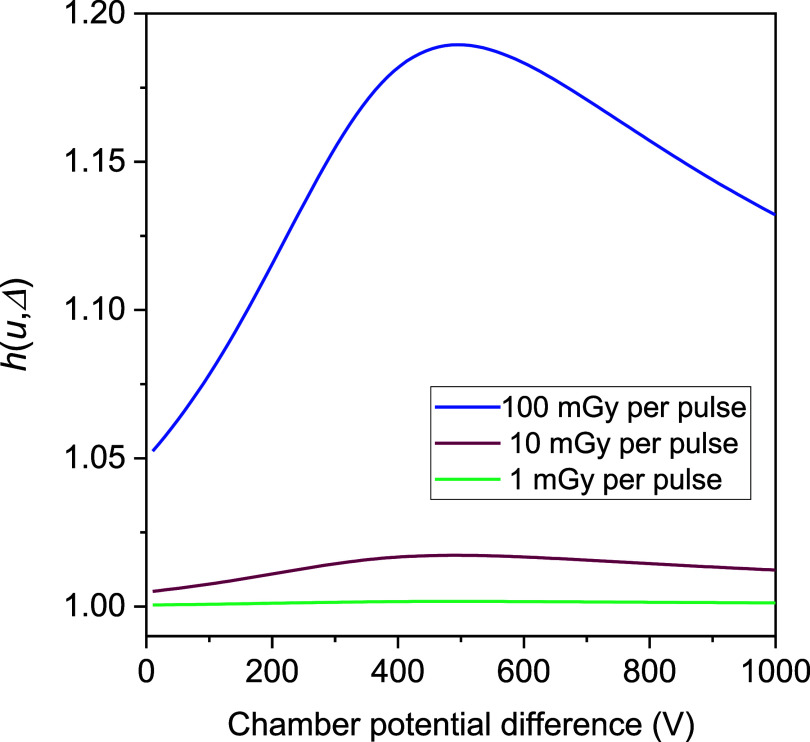
Values of the *h* factor of equation (23) plotted for doses-per-pulse of 1, 10 and 100 mGy and voltages of 100–1000 V. The *h* values are calculated for the cylindrical chamber with inner and outer electrode radii of 0.5 and 2.333 mm, using the transport and recombination coefficients of table 1. *γ* was set to the level in the equivalent parallel plate chamber with the same voltage applied.

The difference between ${f_{{\text{Gauss}}}}$ and Boag’s (1950) $f$ formula for collection efficiency is due to the *h* factor in equation (23). Values of *h* calculated for the cylindrical chamber geometry and the particle transport and recombination coefficients of table 1 are plotted in figure 2 for doses-per-pulse of 1, 10 and 100 mGy and voltages of 100–1000 V. The electron attachment rate-constant, *γ*, was set to the level in the equivalent parallel plate chamber with the same voltage applied. The *h* values are ⩾1, larger for higher doses-per-pulse, and initially rise with increasing voltage before falling again. The initial rise is due to $\gamma $ decreasing with increasing electric field strength. If $\gamma $ is set to a constant value, *h* decreases monotonically with voltage.

Approximate collection efficiency formulae $f_{{\text{cyl}}}^{\prime}$, $f_{{\text{cyl}}}^{{\prime} {\prime}}$ and $f_{{\text{cyl}}}^{{\prime} {\prime} ^{\prime}}$ derived for positively polarized cylindrical chambers have the same forms as $f^{\prime}$, $f^{{\prime} {\prime}}$ and $f^{{\prime} {\prime} ^{\prime}}$ in equations (7)–(9), but again with $\frac{1}{2}\,\left( {r_{{\text{out}}}^2 - r_{{\text{in}}}^2} \right)\,\,{\text{ln}}\left( {{r_{{\text{out}}}}/{r_{{\text{in}}}}} \right)$ replacing *d^2^* in the *u* and *p* terms. The same formulae are obtained for negatively polarized chambers.

### Validation of ${f_{{\text{Gauss}}}}$ excluding *E*-field screening and $\gamma \left( E \right)$ variation

3.2.

Tables 2 and 3 list collection efficiencies calculated from the formulae and computed from the numerical code for parallel plate and cylindrical chambers. Efficiencies computed without accounting for *E*-field screening or *γ*(*E*) variation were the same for positively and negatively polarized cylindrical chambers. They agreed with values obtained from ${f_{{\text{Gauss}}}}$ with a maximum discrepancy of 0.0001 absolute, validating this formula under the conditions for which it was derived. They also agreed with collection efficiencies computed for parallel plate chambers without accounting for *E*-field screening or *γ*(*E*) variation, as expected since the chambers’ dimensions meet the equivalence condition of equation (1).

**Table 2.** Collection efficiencies (CE) calculated for a parallel plate chamber with 2 mm electrode separation, irradiated with doses-per-pulse (DPP) of 0.1–100 mGy. Calculations were made for chamber potential differences of 200 and 400 V, using collection efficiency formulae and a numerical code that included charge screening of the E-field or excluded it (‘idealized’). Relative differences (%) between efficiencies calculated using each method and those from the code with E-field screening are shown in parentheses.

DPP (mGy)	Numerical with *E* screening	Numerical idealized	${f_{{\text{exp}}}}$	$f$	$f^{\prime}$	$f^{{\prime} {\prime}}$	$f^{{\prime} {\prime} ^{\prime}}$
Parallel plate CE at 200 V							
0.1	0.9994	0.9994	0.9994	0.9993	0.9993	0.9994	0.9993
		(0%)	(0%)	(−0.01%)	(−0.01%)	(0%)	(−0.01%)
1	0.9938	0.9939	0.9939	0.9928	0.9934	0.9939	0.9936
		(+0.01%)	(+0.01%)	(−0.10%)	(−0.04%)	(+0.01%)	(−0.02%)
10	0.9412	0.9432	0.9432	0.9336	0.9388	0.9436	0.9412
		(+0.21%)	(+0.21%)	(−0.81%)	(−0.25%)	(+0.25%)	(0%)
100	0.6237	0.6627	0.6627	0.6173	0.6419	0.6647	0.6534
		(+6.25%)	(+6.25%)	(−1.03%)	(+2.92%)	(+6.57%)	(+4.76%)
Parallel plate CE at 400 V							
0.1	0.9998	0.9998	0.9998	0.9996	0.9997	0.9998	0.9998
		(0%)	(0%)	(−0.02%)	(−0.01%)	(0%)	(0%)
1	0.9980	0.9980	0.9980	0.9964	0.9975	0.9983	0.9979
		(0%)	(0%)	(−0.16%)	(−0.05%)	(+0.03%)	(−0.01%)
10	0.9802	0.9808	0.9808	0.9653	0.9760	0.9834	0.9801
		(+0.06%)	(+0.06%)	(−1.52%)	(−0.43%)	(+0.33%)	(−0.01%)
100	0.8168	0.8527	0.8526	0.7514	0.8212	0.8706	0.8475
		(+4.40%)	(+4.38%)	(−8.01%)	(+0.54%)	(+6.59%)	(+3.76%)

**Table 3.** Collection efficiencies (CE) calculated for a cylindrical chamber with 0.5 and 2.333 mm inner and outer electrode radii, irradiated with 0.1–100 mGy doses-per-pulse (DPP). Calculations were made for chamber potential differences of 200 and 400 V, using collection efficiency formulae and a numerical code that included E-field screening and/or *γ(E)* variation or neither (‘idealized’). Relative differences (%) between efficiencies calculated using each method and those from the code with E-field screening and *γ*(E) variation are shown in parentheses.

A. Positive chamber polarity.																		
DPP (mGy)		Numerical with *E* screening & *γ*(*E*)				Numerical with *γ*(*E*)		Numerical idealized		${f_{{\text{Gauss}}}}$		$f$		$f_{{\text{cyl}}}^{\prime}$		$f_{{\text{cyl}}}^{{\prime}{\prime}}$		$f_{{\text{cyl}}}^{{\prime}{{\prime} {\prime}}}$
				Cylindrical CE at +200 V														
0.1		0.9994				0.9994		0.9994		0.9994		0.9993		0.9993		0.9994		0.9993
						(0%)		(0%)		(0%)		(−0.01%)		(−0.01%)		(0%)		(−0.01%)
1		0.9937				0.9938		0.9939		0.9939		0.9928		0.9934		0.9939		0.9936
						(+0.01%)		(+0.02%)		(+0.02%)		(−0.09%)		(−0.03%)		(+0.02%)		(−0.01%)
10		0.9380				0.9423		0.9432		0.9432		0.9336		0.9388		0.9436		0.9412
						(+0.46%)		(+0.55%)		(+0.55%)		(−0.47%)		(+0.09%)		(+0.60%)		(+0.34%)
100		0.5947				0.6585		0.6627		0.6627		0.6173		0.6419		0.6647		0.6534
						(+10.72%)		(+11.43%)		(+11.43%)		(+3.80%)		(+7.94%)		(+11.77%)		(+9.87%)
				Cylindrical CE at +400 V														
0.1		0.9998				0.9998		0.9998		0.9998		0.9996		0.9997		0.9998		0.9998
						(0%)		(0%)		(0%)		(−0.02%)		(−0.01%)		(0%)		(0%)
1		0.9977				0.9978		0.9980		0.9980		0.9964		0.9975		0.9983		0.9979
						(+0.01%)		(0.03%)		(0.03%)		(−0.13%)		(−0.02%)		(+0.06%)		(+0.02%)
10		0.9783				0.9786		0.9808		0.9808		0.9653		0.9760		0.9834		0.9801
						(+0.03%)		(+0.26%)		(+0.26%)		(−1.33%)		(−0.24%)		(+0.52%)		(+0.18%)
100		0.8226				0.8376		0.8527		0.8526		0.7514		0.8212		0.8706		0.8475
						(+1.82%)		(+3.66%)		(+3.65%)		(−8.66%)		(−0.17%)		(+5.84%)		(+3.03%)
B. Negative chamber polarity.																		
DPP (mGy)	Numerical with *E* screening and *γ*(*E*)				Numerical with *γ*(*E*)		Numerical idealized		${f_{{\text{Gauss}}}}$		$f$		$f_{{\text{cyl}}}^{\prime}$		$f_{{\text{cyl}}}^{{\prime}{\prime}}$		$f_{{\text{cyl}}}^{{\prime}{{\prime} {\prime}}}$	
			Cylindrical CE at −200 V															
0.1	0.9994				0.9994		0.9994		0.9994		0.9993		0.9993		0.9994		0.9993	
					(0%)		(0%)		(0%)		(−0.01%)		(−0.01%)		(0%)		(−0.01%)	
1	0.9937				0.9938		0.9939		0.9939		0.9928		0.9934		0.9939		0.9936	
					(+0.01%)		(+0.02%)		(+0.02%)		(−0.09%)		(−0.03%)		(+0.02%)		(−0.01%)	
10	0.9324				0.9423		0.9432		0.9432		0.9336		0.9388		0.9436		0.9412	
					(+1.06%)		(+1.16%)		(+1.16%)		(+0.13%)		(+0.69%)		(+1.20%)		(+0.94%)	
100	0.5215				0.6584		0.6627		0.6627		0.6173		0.6419		0.6647		0.6534	
					(+26.25%)		(+27.07%)		(+27.07%)		(+18.37%)		(+23.09%)		(+27.46%)		(+25.29%)	
			Cylindrical CE at −400 V															
0.1	0.9998				0.9998		0.9998		0.9998		0.9996		0.9997		0.9998		0.9998	
					(0%)		(0%)		(0%)		(−0.02%)		(−0.01%)		(0%)		(0%)	
1	0.9977				0.9978		0.9980		0.9980		0.9964		0.9975		0.9983		0.9979	
					(+0.01%)		(0.03%)		(0.03%)		(−0.13%)		(−0.02%)		(+0.06%)		(+0.02%)	
10	0.9747				0.9786		0.9808		0.9808		0.9653		0.9760		0.9834		0.9801	
					(+0.40%)		(+0.63%)		(+0.63%)		(−0.96%)		(+0.13%)		(+0.89%)		(+0.55%)	
100	0.6889				0.8371		0.8527		0.8526		0.7514		0.8212		0.8706		0.8475	
					(+21.51%)		(+23.78%)		(+23.76%)		(+9.07%)		(+19.20%)		(+26.38%)		(+23.02%)	

### 3.3. Effect of *γ(E)* variation within the cylindrical chamber

Cylindrical chamber collection efficiencies obtained from the numerical code after accounting for *γ*(*E*) variation but not *E*-field screening were slightly lower than those excluding *γ*(*E*) variation and *E* screening (table 3). Differences were greatest at 100 mGy per pulse and 400 V, where efficiencies of 0.8371 and 0.8523 were computed for a negatively polarized chamber with and without *γ*(*E*) variation, a relative difference of 1.8%. At doses-per-pulse ⩽10 mGy, the maximum difference was 0.2%. Computed collection efficiencies accounting for *γ(E)* variation differed marginally between positive and negative chamber polarities, but the maximum difference was only 0.06% relative at 100 mGy per pulse and 400 V chamber voltage.

### 3.4. Effect of E-field screening

**Figure 3.**
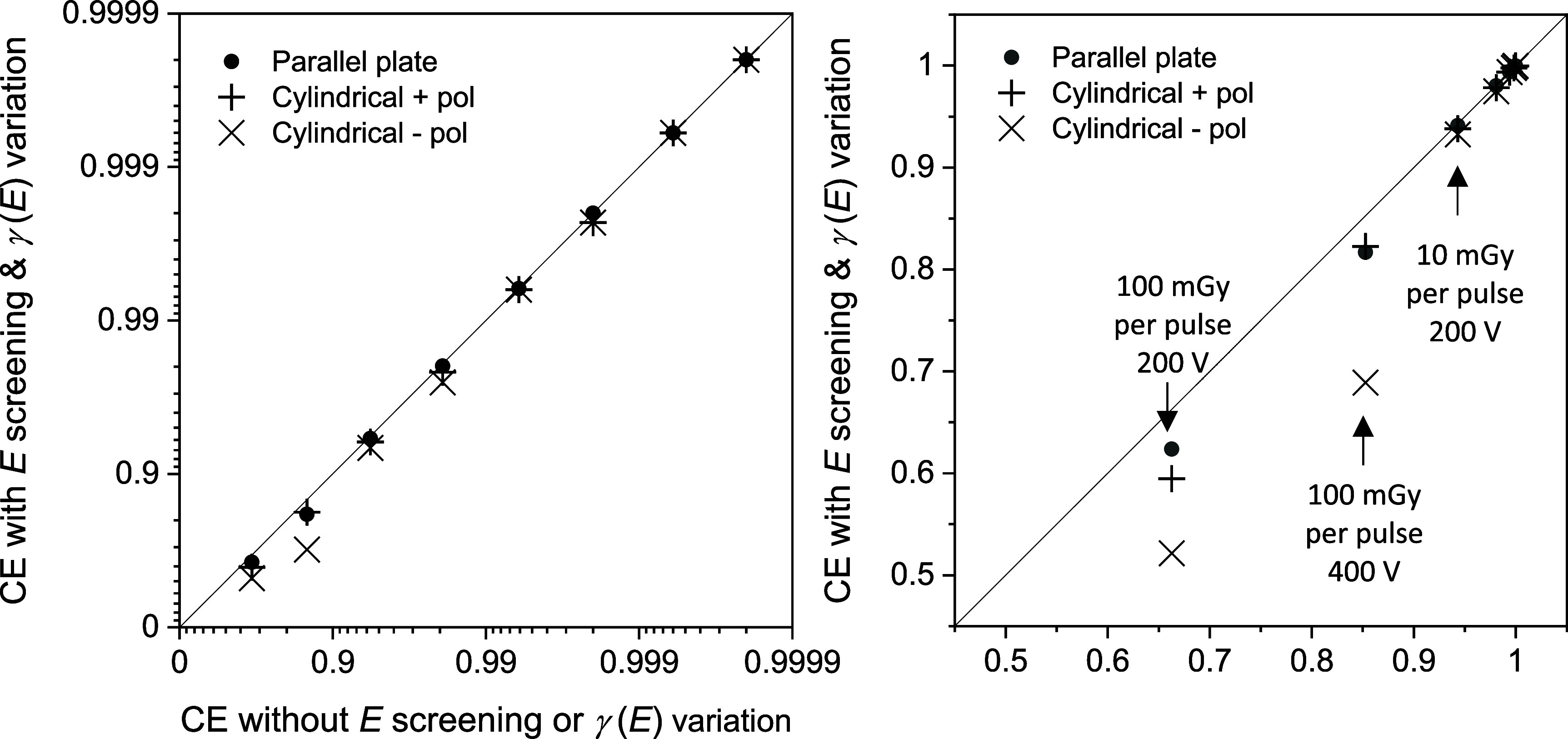
Collection efficiencies (CE) calculated for the parallel plate and cylindrical chambers after accounting for *E*-field screening and *γ*(*E*) variation, plotted on log and linear scales against efficiencies calculated without turning on *E* screening or *γ*(*E*) variation. The line of equality is shown. The lowest collection efficiencies, with the greatest differences between chambers, were calculated for 100 mGy per pulse at chamber potential differences of 200 and 400 V, and for 10 mGy per pulse at 200 V, as indicated in the linear plot.

Tables 2 and 3 also list collection efficiencies computed using the code for parallel plate chambers after accounting for *E*-field screening, and for cylindrical chambers after accounting for both *E* screening and *γ*(*E*) variation. In figure 3 these collection efficiencies are plotted against idealized efficiencies computed without screening or *γ*(*E*) variation.

Screening of the *E*-field reduced collection efficiencies. At 100 mGy per pulse and 200 V, the efficiency computed for the parallel plate chamber after allowing for screening was 5.9% relative lower than the value without screening, and efficiencies calculated for the positively and negatively polarized cylindrical chamber including screening and *γ*(*E*) variation were respectively 10.3% and 21.3% lower than efficiencies calculated without these factors. For doses-per-pulse ⩽10 mGy the effect was much smaller, with a maximum reduction in collection efficiency due to screening of 1.1% relative at 200 V.

### 3.5. The equivalence condition

In Supplementary figure 1, collection efficiencies computed for the cylindrical chamber including *E*-field screening and *γ*(*E*) variation are plotted against efficiencies computed for the parallel plate chamber including screening. At doses-per-pulse ⩽1 mGy, almost equal collection efficiencies were calculated for the two chambers. At 10 mGy per pulse, *E*-field screening and *γ*(*E*) variation still had limited impact on the equivalence of the two chambers. For 200 V chamber voltage, collection efficiencies of 0.9412, 0.9380 and 0.9324 respectively were calculated for the parallel plate chamber and the cylindrical chamber with positive and negative polarity, a maximum relative difference between chambers of 0.9%. For 400 V, the chambers’ collection efficiencies differed by a maximum of 0.6%.

At 100 mGy per pulse, the equivalence between the two chambers broke down after accounting for *E*-field screening and *γ*(*E*) variation. At 200 V the collection efficiencies calculated for the parallel plate chamber and the cylindrical chamber with positive and negative polarity were 0.6237, 0.5947 and 0.5215 respectively, a maximum relative difference between chambers of 19.6%. At 400 V the corresponding efficiencies were 0.8168, 0.8226 and 0.6889, a maximum difference of 18.6%. Since *γ*(*E*) variation alone made only 1.8% difference to the collection efficiency of the cylindrical chamber at 400 V and 100 mGy per pulse (table 3), the breakdown in equivalence is largely due to *E*-field screening.

### 3.6. Which formula describes the computed collection efficiencies best?

Here, collection efficiencies computed using the code are compared with values obtained from the various formulae with parameters determined directly by chamber geometries and the charge transport and recombination coefficients of table 1. Since these coefficients were also used in the code, this gauges how consistent the analytical formulae are with the underlying physics of the computational model, despite the simplifications made in the analytical derivations.

Collection efficiencies computed using the code with *γ*(*E*) variation and *E*-field screening turned off were described exactly by ${f_{{\text{exp}}}}$ and ${f_{{\text{Gauss}}}}$. These efficiencies were overestimated by ⩽2.1% relative by the approximate $f^{{\prime} {\prime}}$ and $f_{{\text{cyl}}}^{^{\prime\prime}}$ formulae, and underestimated by ⩽1.4% by $f^{{\prime} {\prime}}$ and $f_{{\text{cyl}}}^{{\prime} {\prime} ^{\prime}}$, and ⩽3.7% by $f^{\prime}$ and $f_{{\text{cyl}}}^{\prime}$. The $f$ formula, which excludes free electron effects, underestimated the efficiencies by up to 11.9% (tables 2 and 3).

Collection efficiencies computed for the cylindrical chamber accounting for *γ*(*E*) variation but not *E* screening were overestimated by ⩽1.8% by ${f_{{\text{Gauss}}}}$. In fact, ${f_{{\text{Gauss}}}}$ described these efficiencies better than any other formula up to 1 mGy per pulse, and better than $f$, $f_{{\text{cyl}}}^{\prime}$ and $f_{{\text{cyl}}}^{{\prime} {\prime}}$ at all doses-per-pulse. At 10 and 100 mGy per pulse, however, $f_{{\text{cyl}}}^{{\prime} {\prime} ^{\prime}}$ described the efficiencies best, exceeding them by ⩽1.2% (table 3).

Collection efficiencies computed for parallel plate and cylindrical chambers accounting for both *E*-field screening and *γ*(*E*) variation were still generally described best by ${f_{{\text{exp}}}}$ and ${f_{{\text{Gauss}}}}$ for doses-per-pulse up to 1 mGy. At 10 mGy per pulse, efficiencies calculated for the different chambers, voltages and polarities were described best by an assortment of formulae including $f_{{\text{cyl}}}^{\prime}$, $f_{{\text{cyl}}}^{^{\prime\prime\prime}}$ and $f^{^{\prime\prime\prime}}$. At 100 mGy per pulse, *E*-field screening was substantial and the collection efficiencies were described best by the $f$ formula of Boag (1950). This formula did not describe the computed efficiencies particularly well, though, differing from them by up to 8.0% and 18.4% for the parallel plate and cylindrical chambers respectively (tables 2 and 3).

### 3.7. Multi-voltage estimation of collection efficiencies

Supplementary figure 2 shows five sets of collection efficiencies computed using the code, plotted against chamber voltages. One set was calculated for the parallel plate chamber including free electron effects and *E*-field screening (‘Parallel plate’). Another two were calculated for positively and negatively polarized cylindrical chambers accounting for free electron effects, *E*-field screening and *γ*(*E*) variation (‘Cylindrical ± pol’). Another set excluded *E* screening and *γ*(*E*) variation (‘Idealized ${f_{{\text{exp}}}}$’), and a further set additionally excluded free electron effects (‘Boag $f$’). Results for doses-per-pulse of 1, 10 and 100 mGy are graphed separately.

At 1 mGy per pulse the calculated sets of collection efficiencies were all similar except for the ‘Boag $f$’ set, which was lower than the rest because it did not include direct collection of free electrons by the chamber. At 100 mGy per pulse the sets of calculated efficiencies differed far more, driven by greater *E-*field screening at higher doses-per-pulse. For a chamber potential difference of 100 V the screening substantially reduced the *E*-field, leading to slower ion drift speeds and greater recombination. Consequently, all three sets of collection efficiencies that accounted for screening were lower than the set that corresponds to Boag’s $f$ formula, despite this latter set excluding free electron effects. At 10 mGy per pulse the data followed a pattern intermediate between 1 and 100 mGy per pulse.

**Figure 4.**
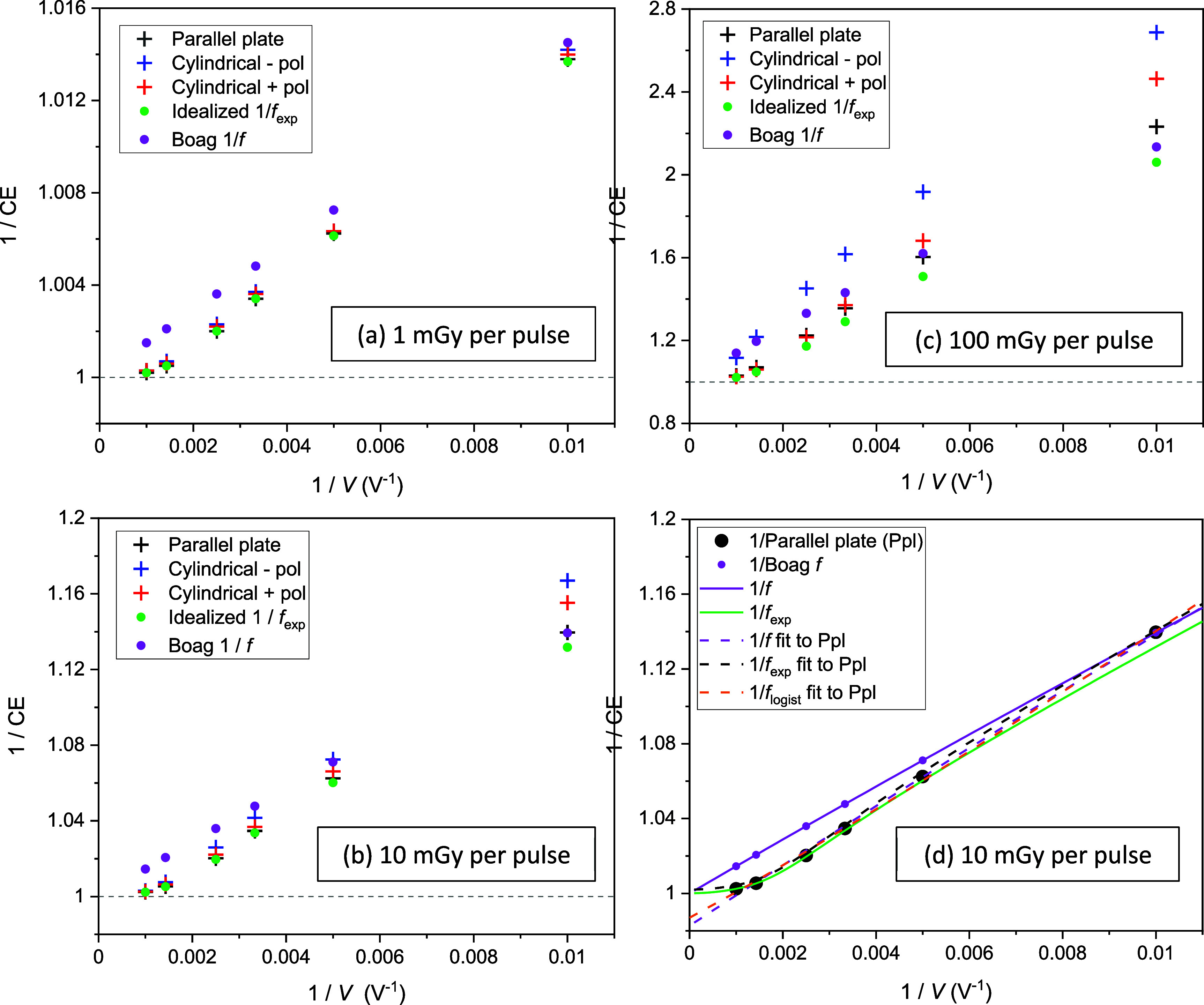
(a)–(c) Jaffé plots showing reciprocals of computed collection efficiencies (CE) versus reciprocals of chamber voltages *V* for doses-per-pulse of 1, 10 and 100 mGy. Sets of efficiencies (‘Parallel plate’, ‘Cylindrical ± pol’) were computed for the parallel plate chamber including free electron effects and *E*-field screening, and for cylindrical chambers including these effects and *γ*(*E*) variation. Another set (‘Idealized *f*_exp_’) excluded *E* screening effects and *γ*(*E*) variation, and a further set (‘Boag $f$’) additionally excluded free electron effects. (d) Reciprocals of ‘Parallel plate’ and ‘Boag $f$’ collection efficiencies computed for 10 mGy per pulse are plotted alongside curves obtained from reciprocals of the *f*(*V*) and *f_exp_* (*V*) formulae with parameter values derived from table 1. Reciprocals of fits of scaled versions of these formulae and the logistic model to the whole 100–1000 V ‘Parallel plate’ dataset are also shown. For brevity, this dataset is referred to in panel (d) as ‘Ppl’.

The same data are presented in figure 4 as Jaffé plots of reciprocals of collection efficiencies versus reciprocals of *V*. For the Boag $f$ set, the plots run roughly linearly through a collection efficiency of unity at (1/*V*) = 0. This result is well known and lies behind a simple experimental method for estimating the ${R_{D,\infty }}$ factor in multi-voltage determination of chamber collection efficiencies, by taking readings at two or more voltages and linearly extrapolating them to (1/*V*) = 0 in a Jaffé plot (Khan 1994, Almond *et al* 1999).

Collection efficiencies that include free electron effects lie along more curved trajectories in Jaffé plots (figure 4). Irrespective of whether these efficiencies account for *E*-field screening or *γ*(*E*) variation, they approach unity at chamber voltages of ∼1000 V, which generate *E*-fields sufficiently high that large fractions of free electrons are collected directly. As a result, ${R_{D,\infty }}$ would be overestimated by linearly extrapolating reciprocals of chamber readings to (1/*V*) = 0, causing collection efficiencies at practical voltages to be underestimated.

Figure 4(d) and supplementary figure 2(d) show fits of scaled nonlinear collection efficiency formulae (${\text{C}}{{\text{E}}_{\infty} } \times {\text{C}}{{\text{E}}_{{\text{model}}}}\left( V \right)$) to collection efficiencies that include free electron effects calculated for 10 mGy per pulse. The fit of the scaled version of Boag’s $f$ formula to collection efficiencies calculated for the parallel plate chamber after accounting for *E* screening and free electrons overestimated the unit collection efficiency at (1/*V*) = 0 by around a factor of 1.02. This is equal to the fitted ${\text{C}}{{\text{E}}_{\infty} }$ factor which, as outlined in the *materials and methods*, describes the relative inaccuracy of the multi-voltage method for determining collection efficiencies. Linear extrapolation of reciprocals of collection efficiencies back to (1/*V*) = 0 overestimated the collection efficiency there to the same extent, since $f$ curves are roughly linear in Jaffé plots.

The fit of the scaled ${f_{{\text{exp}}}}$ formula, which accounts for free electron effects, predicts a collection efficiency much closer to one at (1/*V*) = 0. Screening of the *E*-field had only a limited effect at 10 mGy per pulse, and consequently the plotted collection efficiencies were described quite well by ${f_{{\text{exp}}}}$ despite this formula not accounting for screening. This was even the case when the *u* and *ad* parameters in the formula were calculated directly from the values listed in table 1 and with ${\text{C}}{{\text{E}}_{\infty} }$ set to one, giving the green curves in figure 4(d) and supplementary figure 2(d). When the *u* and *ad* parameters were fitted along with ${\text{C}}{{\text{E}}_{\infty} }$, giving the black dashed curves, the description of collection efficiencies was improved at 100 V where screening has most effect, but slightly worsened at higher voltages.

**Figure 5.**
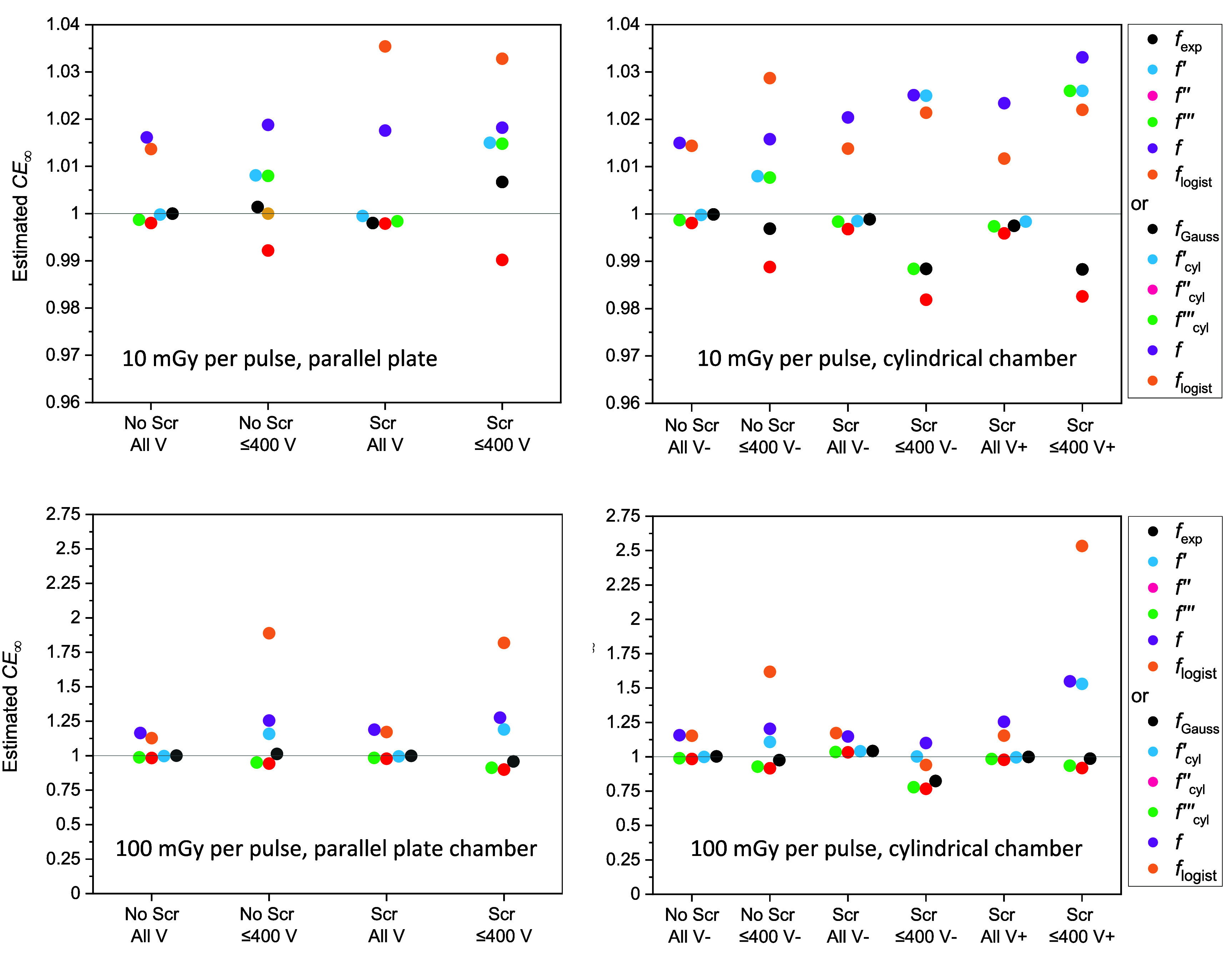
Swarm plots of $C{E_{\infty} }$ values obtained from fits of scaled collection efficiency formulae to efficiencies calculated using the code. The collection efficiencies fitted were calculated with and without *E*-field screening effects (‘Scr’ or ‘No Scr’) for 10 mGy and 100 mGy per pulse delivered to the parallel plate chamber and the cylindrical chamber with positive and negative polarities (‘+’ or’-‘). Formulae were fitted to whole sets of efficiencies calculated for voltages *V* of 100–1000 V and to subsets calculated for *V* ⩽400 V (‘All V’ or ‘⩽ 400 V’).

Figure 5 shows the ${\text{C}}{{\text{E}}_{\infty} }$ values obtained by fitting sets of computed collection efficiencies using scaled $f$, $f^{\prime}$, $f^{{\prime} {\prime}}$, $f^{{\prime} {\prime} ^{\prime}}$ and ${f_{{\text{exp}}}}$ formulae, their cylindrical equivalents and ${f_{{\text{logist}}}}$. The sets of efficiencies fitted were computed for the parallel plate chamber and the cylindrical chamber with positive and negative polarities, irradiated with 10 and 100 mGy per pulse, including or excluding *E* screening effects. For the cylindrical chamber all the computed efficiencies accounted for *γ*(*E*) variation. The ${\text{C}}{{\text{E}}_{\infty} }$ values obtained are further summarized in table 4.

**Table 4.** Summary of the ${\text{C}}{{\text{E}}_{\infty} }$values plotted in figure 5, obtained by fitting scaled collection efficiency formulae to efficiencies computed for parallel plate and cylindrical chambers and doses-per-pulse of 10 and 100 mGy. The fitted efficiencies were computed with and without allowing for the effect of *E*-field screening. Formulae were fitted to all collected efficiencies computed for voltages of 100–1000 V, and to subsets computed for 100–400 V.

Formula	Mean $C{E_{\infty} }$	Median $C{E_{\infty} }$	$C{E_{\infty} }$ range	${\text{RMS}}\left( {{\text{C}}{{\text{E}}_{\infty} } - 1} \right)$ ^a^
	*10* mGy *pulse, all fits to 100–1000* *V datasets and 100–400* *V subsets*			
${f_{{\text{exp}}}}\,$or ${f_{{\text{Gauss}}}}$	0.9983	0.9994	0.9884, 1.0067	0.0036
$f{^{{\prime}}}$ or $f{^{{\prime}}}$	1.0078	1.0039	0.9985, 1.0260	0.0129
$f_{{\text{cyl}}}^{{\prime}}$ or $f_{{\text{cyl}}}^{{\prime} {\prime}}$	0.9922	0.9941	0.9819, 0.9981	0.0098
$f^{{\prime} {\prime} ^{\prime}}$ or ${f^{{\prime} {\prime} ^{\prime}}_{{\text{cyl}}}}$	1.0037	0.9987	0.9884, 1.0260	0.0108
$f$	1.0204	1.0185	1.0150, 1.0331	0.0211
${f_{\text{logist}}}$	1.0194	1.0018	1.0000, 1.0354	0.0219
	*100* mGy *pulse, all fits to 100–1000 V datasets and 100–400 V subsets*			
${f_{\exp }}$or ${f_{{\text{Gauss}}}}$	0.9814	0.9990	0.8237, 1.0423	0.0594
$f^{\prime}$ or ${f^\prime_{{\text{cyl}}}}$	1.1022	1.0213	0.9963, 1.5304	0.1886
$f^{{\prime} {\prime}}$ or ${f^{{\prime} {\prime}}_{{\text{cyl}}}}$	0.9396	0.9606	0.7669, 1.0323	0.0922
$f^{{\prime} {\prime} ^{\prime}}$ or ${f^{{\prime} {\prime} ^{\prime}}_{{\text{cyl}}}}$	0.9489	0.9675	0.7792, 1.0352	0.0836
$f$	1.2299	1.1845	1.1004, 1.5495	0.2187
${f_{{\text{logist}}}}$	1.4578	1.1723	0.9407, 2.5335	0.6572
	*10* mGy *pulse, fits to 100–400 V data subsets*			
${f_{\exp }}$or ${f_{{\text{Gauss}}}}$	0.9983	0.9983	0.9884, 1.0067	0.0062
$f^{\prime}$ or ${f^\prime_{{\text{cyl}}}}$	1.0166	1.0160	1.0080, 1.0260	0.0183
$f^{{\prime} {\prime}}$ or ${f^{{\prime} {\prime}}_{{\text{cyl}}}}$	0.9871	0.9888	0.9819, 0.9922	0.0135
$f^{{\prime} {\prime} ^{\prime}}$ or ${f^{{\prime} {\prime} ^{\prime}}_{{\text{cyl}}}}$	1.0090	1.0080	0.9884, 1.0260	0.0152
$f$	1.0222	1.0188	1.0158, 1.0331	0.0231
${f_{{\text{logist}}}}$	1.0205	1.0220	1.0000, 1.0328	0.0229
	*100* mGy *pulse, fits to 100–400 V data subsets*			
${f_{\exp }}$or ${f_{{\text{Gauss}}}}$	0.9540	0.9757	0.8237, 1.0133	0.0819
$f^{\prime}$ or ${f^\prime_{{\text{cyl}}}}$	1.1223	1.1583	1.0020, 1.5304	0.1388
$f^{{\prime} {\prime}}$ or ${f^{{\prime} {\prime}}_{{\text{cyl}}}}$	0.8888	0.9165	0.7669, 0.9430	0.1313
$f^{{\prime} {\prime} ^{\prime}}$ or ${f^{{\prime} {\prime} ^{\prime}}_{{\text{cyl}}}}$	0.9015	0.9287	0.7792, 0.9509	0.1166
$f$	1.2771	1.2554	1.1004, 1.5495	0.3147
${f_{{\text{logist}}}}$	1.7599	1.8182	0.9407, 2.5335	0.9162

^a^ Root mean square difference between individual${\text{C}}{{\text{E}}_{\infty} }$ values and one.

Typical ${\text{C}}{{\text{E}}_{\infty} }$ values from fits of the scaled Boag (1950) $f$ formula were 1.02 and 1.2 at 10 and 100 mGy per pulse respectively. Values differed little between parallel plate and cylindrical chambers, and were not consistently related to whether the fitted data spanned the whole 100–1000 V range or were limited to chamber voltages ⩽400 V. For fits based on ${f_{{\text{logist}}}}$, $C{E_{\infty} }$ values up to 1.035 and 2.534 were obtained at 10 and 100 mGy per pulse.

$C{E_{\infty} }$ values closer to one were obtained when ${f_{{\text{exp}}}}$, $f^{\prime}$, $f^{{\prime} {\prime}}$, $f^{{\prime} {\prime} ^{\prime}}$, and their cylindrical analogues were used in the fitting. For the sets of collection efficiencies studied, ${f_{{\text{exp}}}}$ and ${f_{{\text{Gauss}}}}$ performed best, achieving mean ${\text{C}}{{\text{E}}_{\infty} }$ values of 0.9983 and 0.9540 at doses-per-pulse of 10 and 100 mGy respectively when the data subsets calculated for voltages ⩽400 V were fitted. Furthermore, root mean square (RMS) differences between unity and the $C{E_{\infty} }$ values obtained from fits to the various sets of collection efficiencies were lower for ${f_{{\text{exp}}}}$ and ${f_{{\text{Gauss}}}}$ than for the other formulae.

At 10 mGy per pulse, $C{E_{\infty} }$ values for fits of the scaled ${f_{{\text{exp}}}}$ and ${f_{{\text{Gauss}}}}$ formulae to the various sets of collection efficiencies ranged from 0.9884 to 1.0067, indicating that errors in collection efficiencies determined using the multi-voltage method should be ⩽1.2%. At 100 mGy per pulse, however, $C{E_{\infty} }$ values for fits of these formulae ranged from 0.8237 to 1.0423, making the approach imprecise.

## 4. Discussion

We have derived a formula ${f_{{\text{Gauss}}}}$ for the collection efficiency of cylindrical ionization chambers by exactly solving a volume recombination model of Boag *et al* (1996) that includes free electron effects. This formula and another, ${f_{{\text{exp}}}}$, similarly derived for parallel chambers have been validated using a numerical code that describes charge transport and recombination. They predict equal collection efficiencies for cylindrical and parallel plate chambers that satisfy the equivalence condition of equation (1), which was originally identified by Boag (1950) for a recombination model that did not include free electrons.

*E*-field screening was not included in the recombination model of Boag *et al* (1996), nor in the derivation of ${f_{{\text{Gauss}}}}\,$ or ${f_{{\text{exp}}}}$, and we therefore used the numerical code to determine by how much collection efficiencies changed when screeing was accounted for. We also calculated collection efficiency changes when the $\gamma \left( E \right)$ variation of the electron attachment rate-constant with the radially varying *E*-field within a cylindrical ionization chamber was factored into the code.

At doses-per-pulse of 0.1 and 1 mGy, *E*-field screening and $\gamma \left( E \right)$ variation made very little difference to calculated collection efficiencies (⩽0.03%) which were therefore still described well by ${f_{{\text{exp}}}}$ and ${f_{{\text{Gauss}}}}$. Thus, at these doses-per-pulse the equivalence condition continued to hold well. At 10 mGy per pulse, *E* screening and $\gamma \left( E \right)$ variation had greater but still limited impact. Collection efficiencies were reduced by ⩽1.1% relative, with efficiencies of the equivalent parallel plate and cylindrical chambers differing by a maximum of 0.9%. At 100 mGy per pulse, *E* screening had much more effect and the chamber equivalence broke down. For 200 V potential difference, collection efficiencies computed for the parallel plate chamber and the cylindrical chamber with positive and negative polarity fell by 5.9%, 10.3% and 21.3% respectively when *E* screening and $\gamma \left( E \right)$ variation were included in calculations for a 200 V chamber potential difference.

The notable difference between the collection efficiencies calculated for the two polarities of the cylindrical chamber at 100 mGy per pulse mirrors the substantially non-unit polarity correction factors of 1.20 and 1.13 reported by Darafsheh *et al* (2021) for IBA CC04 and CC13 cylindrical chambers irradiated at 168 mGy per pulse in a synchro-cyclotron proton beam. The higher efficiency calculated for positive polarity is consistent with results presented by Lapsley (1953) for an argon-filled cylindrical chamber with an outer electrode built from uranium. When Lapsley’s chamber was bombarded with neutrons causing fission fragments to enter the cavity, the chamber voltage needed to achieve a fixed collection efficiency was lower for positive than negative chamber polarity, 120 versus 300 V.

Scaled versions of the collection efficiency formulae were fitted to sets of efficiencies calculated for the parallel plate and cylindrical chambers, including and excluding *E* field screening and $\gamma \left( E \right)\,$variation. Fitted values of CE_∞_ predicted by the various fits describe the relative underestimation of collection efficiencies determined using the multi-voltage method with these formulae.

At 10 mGy and 100 mGy per pulse, fits of the scaled version of Boag’s (1950) $f$ formula gave CE_∞_ values of 1.015–1.033 and 1.100–1.550 respectively. Thus, collection efficiencies determined experimentally using the multi-voltage method with these formulae would be underestimated by 1.5%–3.2% and 9.1%–35.5% respectively at these doses-per-pulse.

Values of CE_∞_ obtained from fits of scaled formulae derived from the model of Boag *et al* (1996) which includes free electron effects were closer to one. For the sets of collection efficiencies studied, fits of ${f_{{\text{exp}}}}$ and ${f_{{\text{Gauss}}}}$ gave CE_∞_ values with the lowest RMS differences from unity. At 10 mGy per pulse, the CE_∞_ values of ${f_{{\text{exp}}}}$ and ${f_{{\text{Gauss}}}}$ fits to efficiencies calculated for voltages of 100–400 V ranged from 0.9884–1.0067. This includes CE_∞_ values from fits to collection efficiencies computed with *E*-field screening and $\gamma \left( E \right)$ variation turned on, effects not included in the derivations of the collection efficiency formulae. According to these results, then, collection efficiencies at 10 mGy per pulse can be determined within an error of 1.2% by analyzing detector readings made at chamber voltages of 100–400 V using the multi-voltage approach with the ${f_{{\text{exp}}}}$ and ${f_{{\text{Gauss}}}}$ formulae.

At 100 mGy per pulse, fits of scaled ${f_{{\text{exp}}}}$ and ${f_{{\text{Gauss}}}}$ formulae also gave CE_∞_ values with the lowest RMS differences from one. However, CE_∞_ values for the fits ranged from 0.824 to 1.013, indicating errors of up to 21.4% in collection efficiencies obtained using multi-voltage methods with these formulae. Use of the other formulae investigated would lead to larger errors.

CE_∞_ values from fits of the scaled empirical formula ${f_{{\text{logist}}}}$ ranged from 1.000 to 1.035 at 10 mGy per pulse, and from 0.941 to 2.531 at 100 mGy. This formula has provided very good fits of ionization chamber measurements made at doses-per-pulse of 10–10^4^ mGy and chamber voltages of 50–300 V (Petersson *et al* 2017). However, its flexible nature might lead to considerable uncertainties in extrapolations of fits to very high voltages.

Unlike ionization chamber readings, the computational collection efficiency data we fitted did not include random measurement uncertainties which could affect the CE_∞_ values obtained. However, uncertainties in ionization chamber readings can be held at low levels, around 0.1% (DeWerd and Smith 2021). And much of the fitted data were calculated after accounting for the effects of *E*-field screening and $\gamma \left( E \right)$ variation. These effects introduce larger differences into the data, up to 23% at 100 mGy per pulse, and since they were not factored into the derivations of the fitted formulae they will have a greater impact on CE_∞_ values.

In practice, ionization chamber collection efficiencies reach values greater than unity at very high electric field strengths, a result of charge amplification caused by free electrons moving at high speeds. In Pinpoint cylindrical chambers with electrode gaps of 0.7–1.15 mm (PTW 31 022 and 31 023) collection efficiencies begin to rise due to charge amplification at voltages of 250–400 V (Bruggmoser *et al* 2018a, 2018b). Similarly, in an experimental parallel plate detector with a 0.25 mm electrode gap, efficiencies start to rise at 375 V (Kranzer *et al* 2022). However, the increases in this voltage range were small, ∼0.3%, and did not depend on dose-per-pulse. Charge amplification can be included in numerical calculations of collection efficiency (Kranzer *et al* 2022, Paz-Martín *et al* 2022) but this is not of interest for our studies. The aim of fitting a recombination model to chamber readings and extrapolating to $V \to \infty $ is to determine the true reading in the absence of both recombination and amplification. Therefore, the ideal model for this purpose should exclude charge amplification and describe recombination rates that fall to zero at high voltages.

In our ion transport code, we assumed that radiation pulses were instantaneous. In reality, pulses have finite durations during which the free electrons and positive ions generated drift to some extent, and this potentially modifies charge distributions and collection efficiencies. However, pulses generated by conventional medical accelerators have durations ⩽5 *μ*s, and differences between efficiencies calculated for instantaneous and 5 *μ*s long pulses were <3% at a high dose-per-pulse of 500 mGy (Paz-Martín *et al* 2022). Diffusion of charge carriers was also ignored in the code and in the analytic solutions. But even if formally ignored, the numerical solution of the equations introduces a pseudo-diffusion that can be close to the physical charge carrier diffusion (Paz-Martin *et al* 2022).

Lastly, it should be noted that in this study the parallel plate and cylindrical ionization chambers were idealized as having electrodes with infinite diameters and lengths respectively. Therefore calculated collection efficiencies do not include the effect of any electric field distortion at the edge of the electrode and guard-ring (Kranzer *et al* 2021).

## 5. Conclusions

We derived a collection efficiency formula ${f_{{\text{Gauss}}}}$ for cylindrical ionization chambers by exactly solving an extended volume recombination model of Boag *et al* (1996) that includes free electron effects. According to this formula and another derived for parallel chambers, ${f_{{\text{exp}}}}$, the two types of ionization chambers should have the same collection efficiencies if they meet the condition $\,\left( {r_{{\text{out}}}^2 - r_{{\text{in}}}^2} \right)\,\,{\text{ln}}\left( {{r_{{\text{out}}}}/{r_{{\text{in}}}}} \right)/2 = \,{d^2}$.

The ${f_{{\text{Gauss}}}}$ formula and the equivalence condition were validated using a numerical code describing ion transport and recombination. The derivations of ${f_{{\text{Gauss}}}}$ and ${f_{{\text{exp}}}}$ did not include *E*-field screening or $\gamma \left( E \right)$ variation. When these effects were factored into the code, calculated collection efficiencies fell by up to 0.03%, 1.1% and 21.3% at doses-per-pulse of 1, 10 and 100 mGy per pulse. The efficiencies calculated at 100 mGy per pulse differed by up to 19.6% relative between parallel plate and cylindrical chambers that met the equivalence condition.

Differences from unity of CE_∞_ values obtained from fits of scaled collection efficiency formulae to efficiencies calculated for 100–400 V chamber voltages correspond to the relative inaccuracies of collection efficiencies determined experimentally by fitting the scaled efficiency formulae to chamber readings made across this voltage range. For the data analyzed, CE_∞_ values closest to one were obtained from fits of the scaled ${f_{{\text{exp}}}}$ and ${f_{{\text{Gauss}}}}$ formulae, being ⩽1.2% and ⩽17.6% from unity at 10 and 100 mGy per pulse respectively. Thus, for the chamber geometries studied, collection efficiencies and corresponding correction factors should be determinable to within 1.2% accuracy up to 10 mGy per pulse using the multi-voltage approach, but with poor accuracy at 100 mGy per pulse.

## Figures and Tables

**Figure A1.**
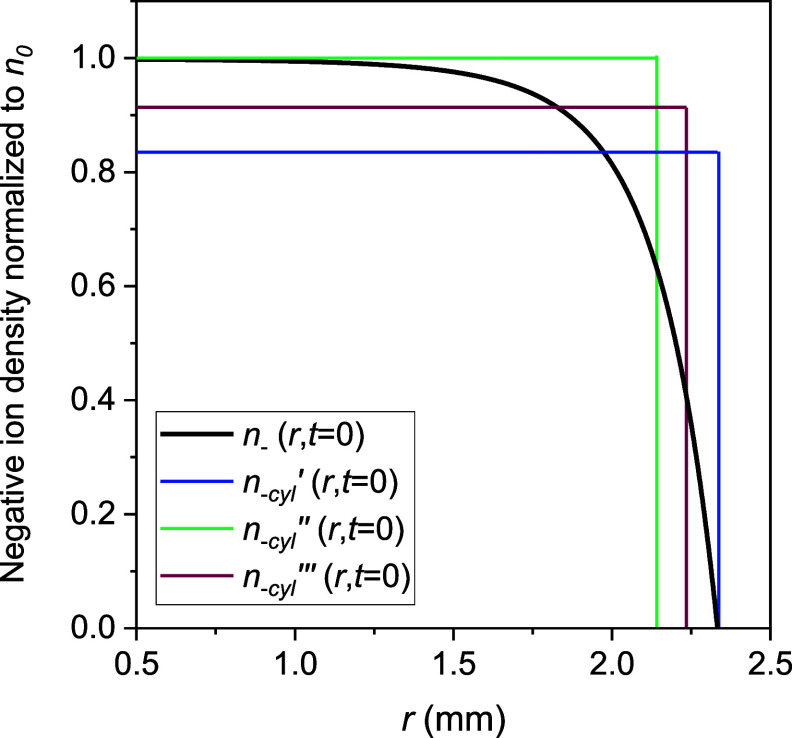
The initial distribution of negative ion densities within a positively polarized cylindrical ionization chamber, $\,{n_ - }\left( {r,t = 0} \right)$, plotted against radial distance *r* from the chamber axis. Also plotted are the approximate distributions $n_{ - ,{\text{cyl}}}^{\prime}$, $n_{ - ,{\text{cyl}}}^{{\prime}{\prime}}$ and $n_{ - ,{\text{cyl}}}^{{\prime}{{\prime} {\prime}}}\,$used to derive the approximate collection efficiency formulae $f_{{\text{cyl}}}^{\prime}$, $f_{{\text{cyl}}}^{{\prime}{\prime}}$ and $f_{{\text{cyl}}}^{{\prime}{{\prime} {\prime}}}$. The densities were calculated for a chamber with 0.5 and 2.333 mm inner and outer electrode radii, using the electron and ion transport and recombination coefficients of table 1. They are plotted relative to the initial uniform positive ion density,$\,{n_0}$.

## Data Availability

All data that support the findings of this study are included within the article (and any supplementary information files).

## Appendix

Key symbols used in the derivation of the ${f_{{\text{Gauss}}}}$ collection efficiency formula are listed below. Numbers of particles are defined per length along the cylindrical chamber axis. Positive and negative ions are abbreviated above as +ve and -ve.

${\text{ }}{r_e}$,$r\,$, original radial locations of electrons and ions respectively

${\text{ }}{r_e}^{\prime}$, $r^{\prime}$, current radial locations of electrons and ions

$\widetilde r$, original radial location of ions that at $r{^{{\prime}}}$ cross ions from $r$ with the opposite sign

${\text{ }}{t_{e,{r_e}}}\left( r \right)$, time at which an electron originating at *r_e_* reaches *r*

${\text{ }}{t_{\tilde r,r}}$, time at which +ve and -ve ions originating at $\tilde r$ and $r$ cross as they drift

${\text{ }}$ *t*_no overlap_, time when the lagging edges of +ve and -ve ion clouds cross

${\text{ }}{n_{e,{r_e}}}\left( t \right)$, density at time *t* of electrons originating at ${r_e}$

${\text{ }}{n_{ \pm ,r}}\left( t \right)$, density at time *t* of +ve ions or -ve ions originating at *r*

${\text{ }}{n_ \pm }\left( {r{^{{\prime}}},t} \right)$, density at time *t* of +ve ions or -ve ions currently located at *r′*

${\text{ }}{\Omega _{ \pm ,r}}\left( t \right) = 2\pi r{n_{ \pm ,r}}\left( t \right)$, density at *t* of ions generated at *r* multiplied by circumference

${\text{ }}{N_0} = {n_0}{ }\pi { }\left( {r_{{\text{out}}}^2 - r_{{\text{in}}}^2} \right)$, initial total number of electrons in a cylindrical chamber

${\text{ }}{N_{ - ,{\text{init}}}}$, initial total number of -ve ions in the chamber

${\text{ }}{N_{ \pm ,{\text{init}},r}}$, total number of $ \pm $ve ions initially located between *r* and the anode

${\text{ }}{N_{ + ,r}}$, total number of +ve ions through which -ve ions generated at *r* pass before reaching the anode

${\text{ }}{R_{ + ,r}}$, difference between ${N_{ + ,{\text{init}},r}}$ and ${N_{ + ,r}}$

${\text{ }}{R_{ - ,r}}$, number of the ${N_{ - ,{\text{init}},r}}\,$-ve ions that recombine before reaching the anode

${\text{ }}{\mathfrak{N}_{ - ,r}}$, number of -ve ions collected that originate between *r_in_* and *r*

${\text{ }}{\mathfrak{N}_{ - ,{\text{tot}}}}$, total number of -ve ions collected

${\text{ }}{\mathfrak{P}_{ + ,{\text{tot}}}}$, number of +ve ions collected that pass through -ve ions

${\text{ }}{\mathfrak{P}_{ + ,{\text{collected}}}}$, total number of +ve ions collected

${\text{ }}\Gamma = \gamma \,{\text{ln}}\left( {{r_{{\text{out}}}}/{r_{{\text{in}}}}} \right)/\left( {2{k_e}V} \right)$, combination of parameters

${\text{ }}{\text{A}} = \frac{{\alpha \,{\text{ln}}\left( {{r_{{\text{out}}}}/{r_{{\text{in}}}}} \right)}}{{\left( {2\pi \left( {{k_1} + {k_2}} \right)V} \right)}}$, combination of parameters

### A1. Free electron transport in the cylindrical geometry

Immediately after a radiation pulse, free electrons are distributed uniformly throughout a cylindrical chamber with density *n*_0_ linearly related to the dose-per-pulse. In chambers with positive polarity the electrons drift inwards towards the anode (figure 1) and at ${r_e}^{\prime}$ off axis they are moving with average speed

\begin{equation*}{v_e}\left( {{r_e}^{\prime}} \right) = \,{k_e}\,E\left( {{r_e}^{\prime}} \right) = \left( {\,{k_e}V/\left( {{r_e}^\prime{\text{ ln}}\left( {{r_{{\text{out}}}}/{r_{{\text{in}}}}} \right)} \right)\,} \right)\,.\end{equation*}

Electrons generated a radial distance *r_e_* from the chamber axis therefore reach the circle of radius ${r_e}^{\prime}$ centred on the chamber axis at a time ${t_{e,{r_e}}}\left( {{r_e}^{\prime}} \right)$ related to *r_e_* via

\begin{align*} {t_{e,{r_e}}}\left( {{r_e}^{\prime}} \right)&amp; = \mathop \int \limits_{{r_e}}^{{r_e}^{\prime}} \frac{{ - {\text{d}}{r_e}^{{\prime} {\prime}}}}{{{v_e}\left( {{r_e}^{{\prime} {\prime}}} \right)}} = \frac{{{\text{ln}}\left( {{r_{{\text{out}}}}/{r_{{\text{in}}}}} \right)}}{{2{k_e}V}}\left( {{r_e}^2 - {r_e}^{{\prime}^2}} \right),\end{align*}

\begin{align*} \kern-9pc{\text{or}}\qquad\qquad\qquad\qquad\qquad\qquad\qquad\,{r_e}&amp; = {{\left( {{ }\frac{{2{k_e}V}}{{{\text{ln}}\left( {{{\text{r}}_{{\text{out}}}}/{r_{{\text{in}}}}} \right)}}{ }{t_{e,{r_e}}}\left( r \right) + {r_e}^{{\prime}^2}} \right)}^{1/2}} .\end{align*}

In the absence of other processes, the density of the electrons originating at *r_e_* would not vary with radius as they drift, because although the electrons converge circumferentially as their distance off-axis decreases, they diverge radially to the same extent due to the varying electric field (Boag 1950). However, over time electrons become attached to oxygen with a rate-constant *γ*. Consequently, on arrival at ${r_e}^{\prime}$ the density ${n_{e,{r_e}}}\left( {{t_{e,{r_e}}}\left( {{r_e}^{\prime}} \right)} \right)$ of free electrons that originate at *r_e_* is less than the initial density *n*_0_ by a factor

\begin{equation*}\frac{{{n_{e,{r_e}}}\left( {{t_{e,{r_e}}}\left( {{r_e}^{\prime}} \right)} \right)}}{{{n_0}}} = {\text{exp}}\left( { - \gamma {t_{e,{r_e}}}\left( {{r_e}^{\prime}} \right)} \right) = {\text{exp}}\left( { - \Gamma \left( {{r_e}^2 - {r_e}^{{\prime}^2}} \right)} \right)\,,\end{equation*}

where $\Gamma = \frac{{\gamma \,{\text{ln}}\left( {{r_{{\text{out}}}}/{r_{{\text{in}}}}} \right)}}{{2{k_e}V}}$. Throughout the appendix the form ${n_r}\left( t \right)$ denotes the density at time *t* of particles generated originally at *r*, whereas *n*(*r’,t*) denotes the density at *t* of particles currently at *r′;*. Thus *n_r_* (*t*) and *n*(*r’,t*) generally differ but are equal when *t* =0 and $r{^{{\prime}}} = r.$

Negative ions are formed at a rate given by the local free electron density multiplied by *γ*, so by time *t* the negative ion density at *r* off-axis is

\begin{equation*}{n_ - }\left( {r,t} \right) = \mathop \int \limits_0^t { }\gamma \;\;{n_{e,{r_e}\left( {r,t^{\prime}} \right)}}\left( {t^{\prime}} \right){\text{ d}}t{^{{\prime}}} = {n_0}{ }\mathop \int \limits_0^t \gamma\;\; {\text{exp}}\left( { - \Gamma \left( {{r_e}{{\left( {r,t{^{{\prime}}}} \right)}^2} - {r^2}} \right)} \right){\text{ d}}t{^{{\prime}}}.\end{equation*}

From (A2) it follows that ${\text{d}}t{^{{\prime}}} = \left\{ {{\text{ln}}\left( {{r_{{\text{out}}}}/{r_{{\text{in}}}}} \right)/\left( {{k_e}V} \right)} \right\}{ }{r_e}{\text{d}}{r_e}$ and so

\begin{equation*}{n_ - }\left( {r,t} \right) = {n_0}\mathop \int \limits_r^{{r_{{\text{max}}}}\left( t \right)} { }2{ }\Gamma { }{r_e}{\text{ exp}}\left( { - \Gamma \left( {r_e^2 - {r^2}} \right)} \right){\text{ d}}{r_e},\end{equation*}

in which *r*_max_(*t*) is the radius of origin of the electrons that cross *r* at time *t*. Integration up to the time when electrons originating at the radius *r*_out_ of the outer electrode arrive at *r* gives

\begin{equation*}{n_ - }\left( r \right) = {n_0}\left( {1 - {\text{exp}}\left( { - \Gamma \left( {r_{{\text{out}}}^2 - {r^2}} \right)} \right)} \right)\,\end{equation*}

which is presented in slightly different form in equation (19) of the *results*. Since no electrons reach *r* from beyond the outer electrode, equation (A6) describes the density distribution of negative ions at the completion of electron transport and before positive or negative ions have moved substantially from their points of origin. This distribution has a Gaussian dependence on *r*. At this early time the total number of negative ions in the chamber per length along its axis is

\begin{equation*}{N_{ - ,{\text{init}}}} = \mathop \int \limits_{{{\text{r}}_{{\text{in}}}}}^{{r_{{\text{out}}}}} 2\pi r{n_ - }\left( r \right){\text{d}}r = {N_0}{ }\left\{ {{ }1 - { }\frac{1}{{{{\Gamma }}\left( {r_{{\text{out}}}^2 - r_{{\text{in}}}^2} \right){ }}}\left( {1 - {\text{exp}}\left( { - \Gamma \left( {r_{{\text{out}}}^2 - r_{{\text{in}}}^2} \right)} \right)} \right){ }} \right\}.\end{equation*}

In equation (A7) *N*_0_ is the initial number of free electrons in the chamber, equal to ${n_0}{ }\pi { }\left( {r_{{\text{out}}}^2 - r_{{\text{in}}}^2} \right)$. Again, *N*_0_ is defined per length along the axis, as particle numbers are throughout the appendix. The initial number of negative ions, ${N_{ - ,{\text{init}}}}$, is equal to the number of uncollected free electrons from which they were formed, given by ${N_0}\left( {1 - p} \right)$ where *p* is the fraction of electrons collected. It therefore follows that

\begin{equation*}p\, = \,\frac{1}{{\Gamma \,\left( {r_{{\text{out}}}^2 - r_{{\text{in}}}^2} \right){ }}}\,\,\left\{ {\,1 - {\text{exp}}\left( { - \Gamma \left( {r_{{\text{out}}}^2 - r_{{\text{in}}}^2} \right)} \right)\,} \right\}\,.\end{equation*}

Repeating this analysis for free electrons travelling outwards in a chamber with negative polarity leads to the negative ion density distribution

\begin{equation*}{n_ - }\left( r \right) = {n_0}\,\left\{ {1 - {\text{exp}}\left( { - \Gamma \left( {{r^2} - \,r_{{\text{in}}}^2} \right)} \right)} \right\},\end{equation*}

with *p* again given by equation (A8).

### A2. Exact solution of the extended recombination model for a cylindrical geometry

Now reset *t* = 0 to the time when all the free electrons have just been collected or become attached to oxygen. Then the initial negative ion distribution $\,{n_{ - ,r}}\left( {t = 0} \right)$ (equivalently ${n_ - }\left( {r,t = 0} \right))$ in a chamber with positive polarity is given by equation (A6) while the initial positive ion distribution ${n_{ + ,r}}\left( {t = 0} \right)$ is uniform with density ${n_0}$.

By time ${t_{\tilde r,r}}\,$later, inward-drifting negative ions initially formed at *r* have reached $r^{\prime}$ off-axis, where they cross outward drifting positive ions initially generated at $\tilde r$ (figure 1). Changes in density as particles drift through the chamber are due to recombination alone, and thus the change in negative ion density between ${t_{\tilde r,r}}$ and ${t_{\tilde r,r}} + dt$ is

\begin{equation*}d{n_{ - ,r}}\left( t \right) = \, - \alpha \,{n_{ - ,r}}\left( {{t_{\tilde r,r}}} \right)\,{n_ + }\left( {r^{\prime},{t_{\tilde r,r}}} \right)\,dt\, = \, - \alpha \,{n_{ - ,r}}\left( {{t_{\tilde r,r}}} \right)\,{n_{ + \widetilde {,r}}}\left( {{t_{\tilde r,r}}} \right)\,dt\,.\end{equation*}

The transport of positive and negative ions is like that of electrons but with mobilities *k*_1_ and *k*_2_ respectively replacing *k_e_*. Consequently,

\begin{equation*}\,\,\,\,\,\,\,\,\,\,{t_{\tilde r,r}} = \frac{{{\text{ln}}\left( {{r_{{\text{out}}}}/{r_{{\text{in}}}}} \right)}}{{2{k_2}V}}\left( {{r^2} - r^{{\prime}^2}} \right) = \frac{{{\text{ln}}\left( {{r_{{\text{out}}}}/{r_{{\text{in}}}}} \right)}}{{2{k_1}V}}\left( {r^{{\prime}^2} - {{\tilde r}^2}} \right) = \,\frac{{{\text{ln}}\left( {{r_{{\text{out}}}}/{r_{{\text{in}}}}} \right)}}{{2\left( {{k_1} + {k_2}} \right)V}}\left( {{r^2} - {{\tilde r}^2}} \right)\,,\end{equation*}

where the third equality follows by eliminating $r^{\prime}$ from the first two. By ${t_{\tilde r,r}} + dt$, the negative ions from $r$ have drifted inward to $r{^{{\prime}}} + {\text{d}}r^{\prime}$ and are crossing positive ions that have drifted out from $\tilde r + d\tilde r$, in which both ${\text{d}}r^{\prime}$ and $d\tilde r$ are negative. Then from (A11)

\begin{equation*}dt = \, - \frac{{{\text{ln}}\left( {{r_{{\text{out}}}}/{r_{{\text{in}}}}} \right)}}{{\left( {{k_1} + {k_2}} \right)V}}\,\tilde r\,d\tilde r,\end{equation*}

and (A10) can be written as

\begin{equation*}\frac{{d{n_{ - ,r}}\left( t \right)}}{{{n_{ - ,r}}\left( t \right)}} = \,\frac{{\alpha \,{\text{ln}}\left( {{r_{{\text{out}}}}/{r_{{\text{in}}}}} \right)}}{{\left( {{k_1} + {k_2}} \right)V}}\,{n_{ + ,\tilde r}}\left( {{t_{\tilde r,r}}} \right)\,\tilde rd\tilde r.\end{equation*}

As they drift inward to the anode, the negative ions that originated at *r* cross positive ions that were generated between *r* and *r*_in_. Integration of (A13) between $\tilde r$ values of *r* and *r*_in_ therefore gives an expression for the fraction of these negative ions that reach the anode

\begin{equation*}\frac{{{n_{ - ,r}}\left( {{t_{{r_{in}},r}}} \right)}}{{{n_{ - ,r}}\left( 0 \right)}} = {\text{exp}}\left( {\,\frac{{ - \alpha \,{\text{ln}}\left( {{r_{{\text{out}}}}/{r_{{\text{in}}}}} \right)}}{{\left( {{k_1} + {k_2}} \right)V}}\,\mathop \int \limits_{{r_{in}}}^r \,{n_{ + \widetilde {,r}}}\left( {{t_{\tilde r,r}}} \right)\,\tilde r\,d\tilde r} \right)\,.\end{equation*}

Now define ${\Omega _{ \pm ,r}}\left( t \right)$ as $2\pi r{n_{ \pm ,r}}\left( t \right),$ so that ${\Omega _{ \pm ,r}}\left( t \right){\text{d}}r$ is the number of positive or negative ions originating between *r* and *r +*d*r* that remain unrecombined by time *t*. Also define ${N_{ + ,r}}$ as the total number of positive ions through which negative ions originating at *r* pass as they drift to the anode, so that

\begin{align*} {N_{ + ,r}}&amp; = \mathop \int \limits_{{r_{{\text{in}}}}}^r {\Omega _{ + ,\tilde r}}\left( {{t_{\tilde r,r}}} \right)d\tilde r \nonumber \\ &amp; = \,\mathop \int \limits_{{r_{{\text{in}}}}}^r {\Omega _{ + ,\tilde r}}\left( {t = 0} \right)d\tilde r - \,\mathop \int \limits_{{r_{{\text{in}}}}}^r {{\Delta }}{\Omega _{ + ,\tilde r}}\left( {{t_{\tilde r,r}}} \right)d\tilde r\,\, = \,\,{N_{ + ,{\text{init}},r}} - {R_{ + ,r}}\,. \end{align*}

In equation (A15) ${{\Delta }}{\Omega _{ + ,\tilde r}}\left( {{t_{\tilde r,r}}} \right)$ is the reduction in ${\Omega _{ + ,\tilde r}}$ between times 0 and ${t_{\tilde r,r}}$, ${N_{ + ,{\text{init}},{\text{r}}}}$ is the total number of positive ions that initially lie between *r* and the anode, and ${R_{ + ,r}}$ is the number of these that have recombined before negative ions originating at *r* pass through them. Then equation (A14) can be rewritten as

\begin{equation*}\frac{{{\Omega _{ - ,r}}\left( {{t_{{r_{in}},r}}} \right)}}{{{\Omega _{ - ,r}}\left( 0 \right)}} = {\text{exp}}\left( {\,\frac{{ - \alpha \,{\text{ln}}\left( {{r_{{\text{out}}}}/{r_{{\text{in}}}}} \right)}}{{2\pi \left( {{k_1} + {k_2}} \right)V}}\mathop \int \limits_{{r_{in}}}^r \,{\Omega _{ + ,\tilde r}}\left( {{t_{\tilde r,r}}} \right)\,d\tilde r} \right) = {\text{exp}}\left( { - {\text A}{N_{ + ,r}}} \right),\end{equation*}

where ${\text A}$ is ${{\alpha \,{\text{ln}}\,\left( {{r_{{\text{out}}}}/{r_{{\text{in}}}}} \right)}} / {{\left( {2\pi \left( {{k_1} + {k_2}} \right)V} \right)}}$.

Next, define ${N_{ - ,{\text{init}},r}}$ as the total number of negative ions that initially lie between the anode and *r*, equal to $\mathop \int \limits_{{r_{in}}}^r {\Omega _{ - r{^{{\prime} {\prime}}}}}\left( 0 \right){\text{ d}}r^{{\prime} {\prime}}$, the integral of ${\Omega _{ - ,r}}\left( 0 \right)$ between *r*_in_ and *r*. And define ${R_{ - ,r}}$ as the number of these ions that recombine before reaching the anode, from which it follows that ${R_{ - ,r}}$ is equal to ${R_{ + ,r}}$. Now $\frac{{{\text{d}}{R_{ - ,r}}}}{{{\text{d}}r}}{\text{d}}r$ is the number of negative ions that originate between *r* and *r*+ *dr* and recombine before reaching the anode, and is therefore given by

\begin{equation*}\frac{{{\text{d}}{R_{ - ,r}}}}{{{\text{d}}r}}{\text{d}}r = { }\left( {{\Omega _{ - ,r}}\left( 0 \right) - {\Omega _{ - ,r}}\left( {{t_{{r_{{\text{in}}}},r}}} \right)} \right){\text{d}}r = { }{\Omega _{ - ,r}}\left( 0 \right){ }\left\{ {{ }1 - {\text{exp}}\left( { - {\text A}{N_{ + ,r}}} \right){ }} \right\}{\text{d}}r.\end{equation*}

From (A15), (A17) and the equality between *R_+,r_* and *R_-,r_*

\begin{equation*}\frac{{{\text{d}}{R_{ - ,r}}}}{{{\text{d}}r}} = {\Omega _{ - r}}\left( 0 \right){ }\left\{ {{ }1 - {\text{exp}}\left( { - {\text A}\left( {{N_{ + ,{\text{init}}}}\left( r \right) - {R_{ - ,r}}} \right)} \right){ }} \right\}.\end{equation*}

Denoting as ${\mathfrak{N}_{ - ,r}}$ the number of negative ions originating between *r*_in_ and *r* that reach the anode, then

\begin{equation*}{\mathfrak{N}_{ - ,r}} = \,{N_{ - ,{\text{init}},r}} - {R_{ - ,r}}\,.\end{equation*}

From (A18) and the differential of (A19) with respect to *r* an equation is obtained that links ${\mathfrak{N}_{ - ,r}}$ to quantities obtained directly from the initial ion distributions

\begin{equation*}\frac{{{\text{d}}{\mathfrak{N}_{ - ,r}}}}{{{\text{d}}r}} = {\Omega _{ - ,r}}\left( 0 \right){\text{ exp }}\left\{ { - {\text A}\left( {{N_{ + ,{\text{init}},r}} - {N_{ - ,{\text{init}},r}} + {\mathfrak{N}_{ - ,r}}} \right){ }} \right\}\end{equation*}

\begin{equation*}\kern-8.8pc{\text{Defining}}\qquad\qquad\qquad\qquad\qquad\;l\left( r \right) = {\Omega _{ - ,r}}\left( 0 \right){\text{ exp}}\left\{ {\, - {\text A}\left( {{N_{ + ,{\text{init}},r}} - {N_{ - ,{\text{init}},r}}} \right)\,} \right\}\,,\end{equation*}

\begin{equation*}{\text{then}}\,\frac{{{\text{d}}{\mathfrak{N}_{ - ,r}}}}{{{\text{d}}r}} = l\left( r \right){\text{ exp}}\left( { - {\text A}{\mathfrak{N}_{ - ,r}}} \right).\end{equation*}

Equation (A22) can be solved by writing

\begin{equation*}m\left( r \right) = {\text{exp}}\left( { - {\text A}{\mathfrak{N}_{ - ,r}}} \right)\end{equation*}

\begin{equation*}\therefore { }\frac{{{\text{d}}m\left( r \right)}}{{{\text{d}}r}} = - {\text A}\frac{{{\text{d}}{\mathfrak{N}_{ - ,r}}}}{{{\text{d}}r}}{\text{exp}}\left( { - {\text A}{\mathfrak{N}_{ - ,r}}} \right) = - {\text A}{ }l\left( r \right){ }{m^2}\left( r \right).\end{equation*}

Integration of (A24) gives

\begin{equation*}\left[ {\frac{1}{m}} \right]_{{r_{{\text{in}}}}}^{{r_{{\text{out}}}}} = { }{\text A}\mathop \int \limits_{{r_{{\text{in}}}}}^{{r_{{\text{out}}}}} l\left( r \right){\text{d}}r,\end{equation*}

and since (1/*m*(*r*)) equals ${\text{exp}}\left( {{{{\text A}}}{\mathfrak{N}_{ - ,r}}} \right)$, and ${\mathfrak{N}_{ - ,{r_{{\text{in}}}}}}$ and ${\mathfrak{N}_{ - ,{r_{{\text{out}}}}}}$are respectively zero and the total number of negative ions reaching the anode, ${\mathfrak{N}_{ - ,{\text{tot}}}}$, then

\begin{equation*}{\mathfrak{N}_{ - ,{\text{tot}}}} = \frac{1}{{{{\text A}}}}{\text{ln}}\left( {1 + {\text A}\mathop \int \limits_{{r_{{\text{in}}}}}^{{r_{{\text{out}}}}} l\left( r \right){\text{d}}r} \right).\end{equation*}

Given the ${n_ - }\left( {r,t = 0} \right)$ distribution of equation (A9) and that ${n_ + }\left( {r,t = 0} \right)$ is uniform with density *n*_0_, it follows that—

\begin{equation*}{\Omega _{ - ,r}}\left( 0 \right) = 2\pi {n_0}{ }r{ }\left\{ {1 - {\text{exp}}\left( { - \Gamma \left( {r_{{\text{out}}}^2 - {r^2}} \right)} \right)} \right\},\end{equation*}

\begin{equation*}{N_{ - ,{\text{init}},r}} = \,\pi {n_0}\left\{ {{r^2} - r_{{\text{in}}}^2 + \frac{1}{\Gamma }{\text{exp}}\left( { - \Gamma \left( {r_{{\text{out}}}^2 - r_{{\text{in}}}^2} \right)} \right) - \frac{1}{\Gamma }{\text{exp}}\left( { - \Gamma \left( {r_{{\text{out}}}^2 - {r^2}} \right)} \right)} \right\}\,,\end{equation*}

\begin{equation*}{N_{ + ,{\text{init}},r}} = \pi {n_0}\left\{ {{r^2} - r_{{\text{in}}}^2} \right\}.\end{equation*}

Substituting these formulae into (A21),

\begin{equation*}l\left( r \right) = {l_1}\left( r \right) - {l_2}\left( r \right),\end{equation*}

where ${l_1}\left( r \right) = {{\text{C}}_1}\,r\,{\text{exp}}\left( { - {{\text{C}}_3}{\text{ exp}}\left( {\Gamma {r^2}} \right)} \right),$

\begin{equation*}{l_2}\left( r \right) = {{\text{C}}_1}{{\text{C}}_2}\,r\,{\text{exp}}\left( {\Gamma {r^2}} \right){\text{ exp}}\left( { - {{\text{C}}_3}{\text{ exp}}\left( {\Gamma {r^2}} \right)} \right)\,,\end{equation*}

and ${C_1} = 2\pi {n_0}{\text{ exp}}\left( { + \frac{{\pi {n_0}{\text A}}}{\Gamma }\,{\text{exp}}\left( { - \Gamma \left( {r_{{\text{out}}}^2 - r_{{\text{in}}}^2} \right)} \right)} \right)\,,$

\begin{equation*}{C_2} = {\text{exp}}\left( { - \Gamma r_{{\text{out}}}^2} \right),\;{C_3} = \frac{{\pi {n_0}{\text A}}}{\Gamma }{\text{ exp}}\left( { - \Gamma r_{{\text{out}}}^2} \right)\,.\end{equation*}

Integration of ${l_1}$ between *r*_in_ and *r*_out_ proceeds by changing variable to $\zeta = {C_3}{\text{exp}}\left( {\Gamma {r^2}} \right)$, for which $\left( {{\text{d}}\zeta /\zeta } \right) = 2\Gamma r{\text{d}}r$. Thus,

\begin{align*} \mathop \int \limits_{{r_{in}}}^{{r_{out}}} {l_1}\left( r \right){\text{d}}r &amp;= \frac{{{C_1}}}{{2\Gamma }}\mathop \int \limits_{\left( {\pi {n_0}{\text A}/\Gamma } \right){\text{ exp}}\left( \Gamma \left( {r_{{\text{in}}}^2 - r_{{\text{out}}}^2} \right) \right)}^{\pi {n_0}{\text A}/\Gamma } { }\frac{{\exp \left( { - \zeta } \right)}}{\zeta }{\text{ d}}\zeta \nonumber\\&amp; = \frac{{{C_1}}}{{2\Gamma }}\left\{ {E_1}\left( \frac{{\pi {n_0}{\text A}}}{\Gamma }{\text{exp}}\left( \Gamma \left( {r_{{\text{in}}}^2 - r_{{\text{out}}}^2} \right) \right) - {E_1}\left( \frac{\pi {n_0}{\text{A}}}{\Gamma } \right) \right) \right\} \end{align*}

where *E_1_* is the exponential integral standard function

\begin{equation*}{E_1}\left( x \right) = \mathop \int \limits_x^\infty \frac{{\exp \left( { - \zeta } \right)}}{\zeta }{\text{d}}\zeta .\end{equation*}

By changing variable from *r* to ${\text{exp}}\left( {\Gamma {r^2}} \right)$, the integral of ${l_2}$ between *r*_in_ and *r*_out_ is $\frac{{{C_1}{C_2}}}{{2{C_3}\Gamma }}\left( {{\text{exp}}\left( { - {C_3}{\text{exp}}\left( {\Gamma r_{{\text{in}}}^2} \right)} \right) - {\text{exp}}\left( { - {C_3}{\text{exp}}\left( {\Gamma r_{{\text{out}}}^2} \right)} \right)} \right)$. Substituting these components of the integral of $l\left( r \right)$ into (A26) gives

\begin{align*} {\mathfrak{N}_{ - ,{\text{tot}}}}&amp;= \frac{1}{{\text{A}}}{\text{In}}\left( {1 + \frac{{{C_1}{\text{A}}}}{{2\Gamma }}\left\{ {{E_1}\left( {\frac{{\pi {n_0}{\text{A}}}}{\Gamma }\exp \left( {\Gamma \left( {r_{{\text{in}}}^2 - r_{{\text{out}}}^2} \right)} \right) - {E_1}\left( {\frac{{\pi {n_0}{\text{A}}}}{\Gamma }} \right.} \right)} \right.} \right) \nonumber \\ &amp; \left. \left. \quad + \frac{{{C_2}}}{{{C_3}}}\left[ {\exp \left( { - {C_3}\exp \left( {\Gamma r_{{\text{out}}}^2} \right)} \right) - \exp \left( { - {C_3}\exp \left( {\Gamma r_{{\text{in}}}^2} \right)} \right)} \right] \right\} \right) .\end{align*}

The collection efficiency ${f_{{\text{Gauss}}}}$ for the Gaussianly-varying negative ion density of equation (A6) can now be obtained by adding to ${\mathfrak{N}_{ - ,{\text{tot}}}}$ the number of free electrons collected, ${n_0}\pi \left( {r_{{\text{out}}}^2 - r_{{\text{in}}}^2} \right)p$, and dividing by the total number of negative electrons initially generated, ${n_0}\pi \left( {r_{{\text{out}}}^2 - r_{{\text{in}}}^2} \right)$. The resulting formula is simplified by extracting a multiplicative factor of $\left\{ {\,1 + \frac{{{C_2}}}{{{C_3}}}\,\left[ {{\text{ exp}}\left( { - {C_{3\,}}{\text{exp}}\left( {\Gamma r_{{\text{out}}}^2} \right)} \right) - {\text{exp}}\left( { - {C_3}{\text{ exp}}\left( {\Gamma r_{{\text{in}}}^2} \right)} \right)\,} \right]\,} \right\}$ from the logarithm term in ${\mathfrak{N}_{ - ,{\text{tot}}}}$ and writing it as a separate additive log term, and further simplified by substituting in the more underlying quantities that make up *C_1_, C_2_* and *C_3_*. Then ${f_{{\text{Gauss}}}}$ works out as

\begin{equation*}{f_{{\text{Gauss}}}} = \frac{1}{u}{\text{ln}}\left\{ {1 + u\,h\left( {u,\Delta } \right)} \right\}\end{equation*}

\begin{equation*}{\text{where}}\;h\left( {u,\Delta } \right)\, = \,\frac{1}{\Delta }{\text{exp}}\left( {\frac{u}{\Delta }} \right)\,\left\{ {{E_1}\left( {\frac{u}{\Delta }{\text{exp}}\left( { - \Delta } \right)} \right) - {E_1}\left( {\frac{u}{\Delta }} \right)} \right\},\end{equation*}

\begin{equation*}u\, = \frac{{{n_0}\alpha }}{{\left( {{k_1} + {k_2}} \right)V}}\,\left\{ {\,\left( {r_{{\text{out}}}^2 - r_{{\text{in}}}^2} \right)\,\,{\text{ln}}\left( {{{\text{r}}_{{\text{out}}}}/{r_{{\text{in}}}}} \right)/2\,} \right\}\,,\end{equation*}

\begin{equation*}{\text{and }}\Delta = \,\frac{\gamma }{{{k_e}V}}\left\{ {\,\left( {r_{{\text{out}}}^2 - r_{{\text{in}}}^2} \right){\text{ ln}}\left( {{r_{{\text{out}}}}/{r_{{\text{in}}}}} \right)/2\,} \right\}\,.\end{equation*}

For negative chamber polarity, the collection efficiency can be derived via equations (A10)–(A26) above except that now the positive ions are moving inwards. The result is an equation for the number of positive ions collected, ${\mathfrak{N}_{ + ,{\text{tot}}}}$, that has the same form as (A26), but with ${N_{ - ,{\text{init}},r}}$ and ${N_{ + ,{\text{init}},r}}$ swapping places in $l\left( r \right)$, and ${\Omega _{ + ,r}}\left( 0 \right)$ replacing ${\Omega _{ - ,r}}\left( 0 \right)$. Solving this equation for ${\mathfrak{N}_{ + ,{\text{tot}}}}$ and dividing by the initial number of positive ions ${n_0}\pi \left( {r_{{\text{out}}}^2 - r_{{\text{in}}}^2} \right)$ gives the collection efficiency. Interestingly, it is the same as for positive chamber polarity, again given by equations (A36)–(A39).

### A3. Approximate solution $f_{{\text{cyl}}}{{\prime}}$ of the extended model for a cylindrical geometry

Consider a cylindrical chamber with positive polarity and the exact negative ion distribution of equation (A6) replaced by an approximate initial distribution

\begin{equation*}n_{ - ,{\text{cyl}}}^{\prime}\left( {r,t = 0} \right) = \,\left( {1 - p} \right)\,{n_0},\end{equation*}

where *p* is the fraction of free electrons collected, described by (A8). The exact distribution ${n_ - }$ is compared in figure A1 to the approximate distributions $n_{ - ,{\text{cyl}}}^{\prime}$, $n_{ - ,{\text{cyl}}}^{{\prime}{\prime}}$ and $n_{ - ,{\text{cyl}}}^{{\prime}{{\prime} {\prime}}}\,$used in the derivation of $f_{{\text{cyl}}}^{\prime}$, $f_{{\text{cyl}}}^{{\prime}{\prime}}$ and $f_{{\text{cyl}}}^{{\prime}{{\prime} {\prime}}}$. Densities were calculated for a cylindrical chamber with 0.5 and 2.333 mm inner and outer electrode radii, using the transport and recombination coefficients of table 1.

Since $n_{ - ,{\text{cyl}}}^{\prime}\left( {r,t = 0} \right)$ and the initial distribution of positive ions are both uniform, rates of change of ion densities within the overlap region between negative and positive ions are also uniform, as are the ion densities ${n_ - }\left( t \right)\,$and ${n_ + }\left( t \right)\,$at later times. The rates of change are given by

\begin{equation*}\frac{{{\text{d}}{n_ - }\left( t \right)}}{{{\text{d}}t}} = \frac{{{\text{d}}{n_ + }\left( t \right)}}{{{\text{d}}t}} = - \alpha {n_ - }\left( t \right){n_ + }\left( t \right).\end{equation*}

Because these rates are equal the difference between ${n_ - }\left( t \right)\,$and ${n_ + }\left( t \right)\,$is constant, at a level *pn_0_* since the initial density of positive ions is *n_0_*. Therefore

\begin{equation*}\frac{{{\text{d}}{n_ - }\left( t \right)}}{{{\text{d}}t}} = - \alpha { }{n_ - }\left( t \right){ }\left( {{n_ - }\left( t \right) + p{n_0}} \right),\,\,\frac{{{\text{d}}{n_ + }\left( t \right)}}{{{\text{d}}t}} = - \alpha { }\left( {{n_ + }\left( t \right) - p{n_0}} \right){ }{n_ + }\left( t \right),\end{equation*}

which integrate to

\begin{equation*}{n_ - }\left( t \right) = \frac{{\left( {1 - p} \right)p{n_0}}}{{{\text{exp}}\left( {p\alpha {n_0}t} \right) - \left( {1 - p} \right)}},\;\,{n_ + }\left( t \right) = \frac{{p{n_0}}}{{1 - \left( {1 - p} \right){\text{exp}}\left( { - p\alpha {n_0}t} \right)}}.\end{equation*}

By time *t* the lagging edge of negative ions formed at the outer edge of the chamber, *r*_out_, has drifted inward to a radius ${r_{{\text{lag}}, - }}^{\prime}\left( t \right)$. At this distance off-axis the positive and negative ions are moving past each other with speeds ${v_ + }\left( {{r_{{\text{lag}}, - }}^{\prime}\left( t \right)} \right)$ and ${v_ - }\left( {{r_{{\text{lag}}, - }}^{\prime}\left( t \right)} \right)$, and the number of positive ions crossing the lagging edge between *t* and *t*+ d*t* is therefore

\begin{align*} {{\dot {\mathfrak{P}}}_ + }\left( t \right)dt\,&amp; = \,2\pi \,{r_{{\text{lag}}, - }}^{\prime}\left( t \right)\,{n_ + }\left( t \right)\,\left\{ {\,{v_ - }\left( {{r_{{\text{lag}}, - }}^{\prime}\left( t \right)} \right) + {v_ + }\left( {{r_{{\text{lag}}, - }}^{\prime}\left( t \right)} \right)\,} \right\}\,dt\, \nonumber \\ &amp; = \,2\pi \,{r_{{\text{lag}}, - }}^{\prime}\left( t \right)\,{n_ + }\left( t \right)\,\left( {{k_1} + {k_2}} \right)\,E\left( {\,{r_{{\text{lag}}, - }}^{\prime}\left( t \right)} \right)dt\,\, = \,\,\,\frac{{2\pi \,\left( {{k_1} + {k_2}} \right)\,V}}{{{\text{ln}}\left( {{r_{{\text{out}}}}/{\text{in}}} \right)\,}}{n_ + }\left( t \right)\,dt. .\end{align*}

All these positive ions go on to be collected since no further negative ions lie between them and the cathode. Thus, the total number of positive ions collected is

\begin{equation*}{\mathfrak{P}_{ + ,{\text{tot}}}} = \mathop \int \limits_0^{{t_{{\text{no overlap}}}}} {\text{ }}{\dot {\mathfrak{P}}_ + }\left( t \right){\text{ d}}t = \frac{\alpha }{{\text A}}\mathop \int \limits_0^{{t_{{\text{no overlap}}}}} {\text{ }}{n_ + }\left( t \right){\text{d}}t,\end{equation*}

where *t*_no overlap_ is the time at which the lagging edges of the positive and negative ion clouds formed at *r*_out_ and *r*_in_ cross and no overlap remains. Substituting (A43) into (A45)

\begin{equation*}{\mathfrak{P}_{ + ,{\text{tot}}}} = \frac{{\alpha p{n_0}}}{{\text A}}{\text{ }}\mathop \int \limits_0^{{t_{{\text{no overlap}}}}} {\text{ }}\frac{1}{{1 - \left( {1 - p} \right){\text{ exp}}\left( { - \alpha p{n_0}t} \right)}}{\text{d}}t.\end{equation*}

Changing variable to $\xi = {\text{exp}}\left( { - \alpha p{n_0}t} \right)$ and writing $\alpha p{n_0}{t_{{\text{no overlap}}}}$ as *T*,

\begin{align*} {\mathfrak{P}_{ + ,{\text{tot}}}}&amp; = \frac{1}{{\text A}}{\text{ }}\mathop \int \limits_{{\text{exp}}\left( { - T} \right)}^1 {\text{ }}\frac{1}{{\xi \left( {1 - \left( {1 - p} \right){{\xi }}} \right)}}{\text{d}}\xi = \frac{1}{{\text{A}}{\text{ }}\left[ {{\text{ ln}}\left( {\frac{\xi }{{1 - \left( {1 - p} \right)\xi }}} \right){\text{ }}} \right]_{{\text{exp}}\left( { - T} \right)}^1} \nonumber\\ &amp;= \frac{1}{{\text A}}{\text{ ln}}\left( {1 + \frac{{{\text{ exp}}\left( { + T} \right) - 1}}{p}} \right) .\end{align*}

From (A11), ${t_{{\text{no overlap}}}} = \,\frac{{{\text{ln}}\left( {{r_{{\text{out}}}}/{r_{{\text{in}}}}} \right)}}{{2\left( {{k_1} + {k_2}} \right)V}}\left( {r_{{\text{out}}}^2 - r_{{\text{in}}}^2} \right)\,.$

Substituting this into (A47) and dividing by the total initial number of positive ions ${n_0}\pi \left( {r_{{\text{out}}}^2 - r_{{\text{in}}}^2} \right)$ gives the approximate collection efficiency formula

\begin{equation*}f^{\prime_{cyl}} = \frac{1}{u}\,{\text{ln}}\left\{ {1 + \frac{{{\text{ exp}}\left( { + pu} \right)\, - \,1}}{p}} \right\}\end{equation*}

with *u* defined as in (A38). When the chamber polarity is negative the drift directions of the positive and negative ions are reversed, but the same set of equations and the same *f′* formula are obtained.

### A4. Approximate solution $f_{{\text{cyl}}}^{^{\prime\prime}}$ of the extended model

Now consider the cylindrical chamber with positive polarity and another approximation to the initial negative ion distribution

\begin{align*} n_{ - ,{\text{cyl}}}^{{\prime} {\prime}}\left( r \right)&amp; = { }{n_{0{ }}},{\text{ }}{r_{{\text{in}}}} &lt; r \le {r_{ - ,{\text{edge}}}}\nonumber\\&amp; = 0,{ }{r_{ - ,{\text{edge}}}} &lt; r &lt; {r_{{\text{out}}}}\end{align*}

\begin{equation*}\kern-11.5pc{\text{where}}\qquad\qquad\qquad\qquad\qquad\qquad\,\,\left( {r_{ - ,{\text{edge}}}^2 - r_{{\text{in}}}^2} \right) = \left( {1 - p} \right){ }\left( {r_{{\text{out}}}^2 - r_{{\text{in}}}^2} \right).\end{equation*}

In the overlap region the densities of positive and negative ions are equal and their rates of change are

\begin{equation*}\frac{{{\text{d}}{n_ - }\left( t \right)}}{{{\text{d}}t}} = \frac{{{\text{d}}{n_ + }\left( t \right)}}{{{\text{d}}t}} = - \alpha {n_ - }\left( t \right){n_ + }\left( t \right) = - \alpha {n_ - }\left( t \right){n_ - }\left( t \right) = - \alpha {n_ + }\left( t \right){n_ + }\left( t \right),\end{equation*}

which integrate to

\begin{equation*}{n_ - }\left( t \right) = {n_ + }\left( t \right) = {n_0}/\left( {1 + {n_0}\alpha t} \right)\,.\end{equation*}

Following the method used to find $f_{{\text{cyl}}}^{\prime}$, the same formula (A45) is obtained for ${\mathfrak{P}_{ + ,{\text{tot}}}}$ in terms of ${n_ + }\left( t \right)$. However, ${n_ + }\left( t \right)$ is now defined by (A52) and *t*_no overlap_ is the time at which the lagging edges of the positive and negative ion clouds formed at ${r_{{\text{in}}}}$ and ${r_{ - {\text{edge}}}}$ cross, given by

\begin{equation*}{t_{{\text{no overlap}}}} = \,\frac{{{\text{ln}}\left( {{r_{{\text{out}}}}/{r_{{\text{in}}}}} \right)}}{{2\left( {{k_1} + {k_2}} \right)V}}\left( {r_{ - ,{\text{edge}}}^2 - r_{{\text{in}}}^2} \right) = \,\frac{{\left( {1 - p} \right){\text{ ln}}\left( {{r_{{\text{out}}}}/{r_{{\text{in}}}}} \right)}}{{2\left( {{k_1} + {k_2}} \right)V}}\left( {r_{{\text{out}}}^2 - r_{{\text{in}}}^2} \right)\,.\end{equation*}

Therefore

\begin{equation*}{\mathfrak{P}_{ + ,{\text{tot}}}}\,\, = \,\,\frac{{\alpha {n_0}}}{A}\,\mathop \int \limits_0^{{t_{{\text{no overlap}}}}} \,\frac{1}{{1 + \alpha {n_0}t}}dt\,\, = \,\frac{1}{A}{\text{ln}}\left( {1 + \alpha \,{n_0}\,{t_{{\text{no overlap}}}}} \right),\end{equation*}

where ${\mathfrak{P}_{ + ,{\text{tot}}}}$ describes the number of positive ions that originate between ${r_{{\text{in}}}}$ and ${r_{ - ,{\text{edge}}}}$ and pass through some of the negative ion cloud and are collected at the cathode. Further positive ions formed between ${r_{ - ,{\text{edge}}}}$ and ${r_{{\text{out}}}}$ also reach the cathode but without passing through any of the negative ion cloud. Adding these to ${\mathfrak{P}_{ + ,{\text{tot}}}}$ gives the total number of positive ions collected

\begin{equation*}{\mathfrak{P}_{ + ,{\text{collected}}}} = \pi \left( {r_{{\text{out}}}^2 - r_{ - {\text{edge}}}^2} \right){ }{n_0} + \frac{1}{A}{\text{ln}}\left( {1 + \alpha { }{n_0}{ }{t_{{\text{no overlap}}}}} \right).\end{equation*}

Substituting *t*_no overlap_ from (A53) and dividing by the total number of positive ions initially formed, $\pi \left( {r_{{\text{out}}}^2 - r_{{\text{in}}}^2} \right){n_0}$, then gives

\begin{equation*}f_{{\text{cyl}}}^{^{\prime\prime}} = p + \frac{1}{u}{\text{ln}}\left\{ {1 + u\left( {1 - p} \right)} \right\}\end{equation*}

with *u* defined by (A38).

For negative chamber polarity, charge flow directions are reversed and the initial negative ion distribution is approximated as *n*_0_ between ${r_{ - ,{\text{edge}}}}$ and ${r_{{\text{out}}}}$, and zero elsewhere, and now $( {r_{{\text{out}}}^2 - r_{ - ,{\text{edge}}}^2} ) = ( {1 - p} )\,\,\left( {r_{{\text{out}}}^2 - r_{{\text{in}}}^2} \right)$. When (A54) and (A55) are adjusted to account for the resulting change in *t*_no overlap_, and to reflect that now positive ions originating in the region ${r_{{\text{in}}}}\,$ to ${r_{ - ,{\text{edge}}}}$ reach the cathode without passing through the negative ion cloud, the $f_{{\text{cyl}}}^{{\prime}{\prime}}$ formula of (A56) is again obtained.

### A5. Approximate solution $f_{{\text{cyl}}}^{{\prime\prime} {\prime}}$ of the extended model

Consider once more the cylindrical chamber with positive polarity, now with the exact negative ion distribution replaced by the approximate initial distribution

\begin{align*} n_{ - ,{\text{cyl}}}^{{\prime\prime} {\prime}}\left( r \right)&amp; = { }\left( {1 - \lambda } \right){ }{n_{0{ }}},{\text{ }}{r_{{\text{in}}}} &lt; r \le {r_{ - ,{\text{edge}}}} \\ &amp; = 0,{ }{r_{ - ,{\text{edge}}}} &lt; r &lt; {r_{{\text{out}}}} \end{align*}

\begin{equation*}\kern-12.7pc{\text{where }}\qquad\qquad\qquad\qquad\qquad\;\left( {r_{ - ,{\text{edge}}}^2 - r_{{\text{in}}}^2} \right) = \left( {1 - \lambda } \right)\,\,\left( {r_{{\text{out}}}^2 - r_{{\text{in}}}^2} \right),\end{equation*}

\begin{equation*}{\text{and }}\,\,\,\,{\left( {1 - \lambda } \right)^2} = \left( {1 - p} \right)\end{equation*}

so that the initial number of negative ions is lower than that of positive ions by a factor of $\left( {1 - p} \right)$, and the initial density of the negative ions and volume in which they are located are both reduced by factors of $\sqrt {\left( {1 - p} \right)} $ compared to the positive ion distribution.

Equations (A41)–(A47) obtained in the derivation of $f_{{\text{cyl}}}^{{\prime}}$ continue to apply, except that the *p* factor appearing in them is replaced by *λ*, and *t*_no overla*p*_ denotes the time at which the lagging edge of the positive ion distribution crosses the negative ion lagging edge which now originates at ${r_{ - {\text{edge}}}}$ rather than *r*_out_. From (A11) and (A58)

\begin{equation*}{t_{{\text{no overlap}}}} = \,\frac{{{\text{ln}}\left( {{r_{{\text{out}}}}/{r_{{\text{in}}}}} \right)}}{{2\left( {{k_1} + {k_2}} \right)V}}\left( {r_{ - ,{\text{edge}}}^2 - r_{{\text{in}}}^2\,} \right) = \,\frac{{\left( {1 - \lambda } \right){\text{ ln}}\left( {{r_{{\text{out}}}}/{r_{{\text{in}}}}} \right)}}{{2\left( {{k_1} + {k_2}} \right)V}}\left( {r_{{\text{out}}}^2 - r_{{\text{in}}}^2} \right)\,.\end{equation*}

Making these changes in (A47) gives

\begin{equation*}{\mathfrak{P}_{ + ,{\text{tot}}}} = \frac{1}{{\text A}}\,\left\{ {{\text{ln}}\left( {1 + \frac{{{\text{ exp}}\left( { + T} \right)\, - \,1}}{\lambda }} \right)} \right\} = \frac{1}{{\text A}}\,\left\{ {{\text{ln}}\left( {1 + \frac{{{\text{ exp}}\left( { + \lambda \left( {1 - \lambda } \right)u} \right)\, - \,1}}{\lambda }} \right)} \right\}\,.\end{equation*}

Adding to ${\mathfrak{P}_{ + ,{\text{tot}}}}$ the positive ions that are generated between *r*_out_ and ${r_{ - {\text{edge}}}}$ and reach the cathode without passing through any negative ions, the total number of positive ions collected is

\begin{equation*}{\mathfrak{P}_{ + ,{\text{collected}}}} = \,\pi \left( {r_{{\text{out}}}^2 - r_{{\text{edge}}}^2} \right)\,{n_0} + \frac{1}{{\text A}}\,\left\{ {{\text{ln}}\left( {1 + \frac{{{\text{ exp}}\left( { + \lambda \left( {1 - \lambda } \right)u} \right)\, - \,1}}{\lambda }} \right)} \right\}\,.\end{equation*}

Dividing by the initial number of positive ions $\pi \left( {r_{{\text{out}}}^2 - r_{{\text{in}}}^2} \right){n_0}$ then gives the collection efficiency

\begin{equation*}f_{{\text{cyl}}}^{{\prime\prime} {\prime}} = \lambda + \frac{1}{u}\,{\text{ln}}\left\{ {1 + \frac{{{\text{ exp}}\left( { + \lambda \left( {1 - \lambda } \right)u} \right)\, - \,1}}{\lambda }} \right\}\,.\end{equation*}

For negative chamber polarity, the initial negative ion distribution is approximated as $\left( {1 - \lambda } \right)\,{n_{0\,}}$between *r*_-,edge_ and *r*_out_, and zero elsewhere, and now

\begin{equation*}\left( {r_{{\text{out}}}^2 - r_{ - ,{\text{edge}}}^2} \right) = \left( {1 - \lambda } \right)\,\,\left( {r_{{\text{out}}}^2 - r_{{\text{in}}}^2} \right)\,.\end{equation*}

Equations (A41)–(A47) continue to apply, but with *λ* again replacing *p* and the lagging edge of the positive ion distribution originating at *r*_out_ rather than *r*_in_. No change results in the expressions for *t*_no overlap_ and ${\mathfrak{P}_{ + ,{\text{tot}}}}$ in (A60) and (A61). The number of additional positive ions originating outside the initial negative ion cloud is also unchanged, and the collection efficiency is again described by the $f_{{\text{cyl}}}^{{\prime\prime} {\prime}}$ formula of (A63).
